# Tool antibody fragments reveal multiple conformations of the rhodopsin-Gi signaling complex

**DOI:** 10.1016/j.bpj.2025.09.044

**Published:** 2025-09-29

**Authors:** Filip Pamula, Oliver Tejero, Jonas Mühle, Ralf Thoma, Gebhard F.X. Schertler, Jacopo Marino, Ching-Ju Tsai

**Affiliations:** 1 Laboratory of Biomolecular Research, Paul Scherrer Institute, Villigen, Switzerland; 2 Department of Biology, ETH Zürich, Zürich, Switzerland; 3 Pharma Research and Early Development (pRED), Roche Innovation Center Basel, F. Hoffmann-La Roche Ltd, Basel, Switzerland

## Abstract

Antibody Fab fragments are widely used protein binders that assist in structural studies of G-protein-coupled receptor (GPCR) signaling complexes. Expanding the repertoire of such binders to target distinct components of the signaling complex offers opportunities to probe conformational regulation and dynamics. Here, we report the biochemical and cryo-EM characterization of two Fab fragments, Fab79 and Fab13, raised against the rhodopsin-Gαiβγ complex. Fab79 binds to the flexible α-helical domain (AHD) of the Gαi subunit and prevents complex dissociation in the presence of the nonhydrolyzable GTP analog, GTPγS, likely by hindering AHD closure, a step necessary for complex dissociation. In contrast, Fab13 binds rigidly to Gβ without directly contacting Gα or the receptor. These findings show that Fab79 and Fab13 reveal functionally relevant conformational states of G-protein activation and serve as practical tools to stabilize or modulate GPCR signaling complexes.

## Significance

Antibody fragments are widely used to capture intermediate states of GPCR-G-protein complexes for structural studies, yet their impact on signaling mechanisms remains underexplored. This study characterizes two Fab fragments that bind distinct G-protein subunits and modulate conformational states relevant to complex stability and activation. These tools provide new opportunities to investigate dynamic aspects of GPCR and G-protein signaling beyond structural determination.

## Introduction

G-protein-coupled receptors (GPCRs) and the signaling complexes they form with G-proteins and arrestins are central to pharmacology and drug discovery (1). Approximately 34% of FDA-approved drugs target GPCRs, making it essential to understand the molecular basis of GPCR signaling. This knowledge contributes not only to structure-based drug design but also to rationalizing the effect of biased agonism (2,3). Such insights can support the development of small molecules that selectively activate desired cellular pathways, potentially reducing side effects for patients.

GPCR signaling elicits a variety of biological responses. It consists of an ensemble of fast and highly dynamic events involving three classes of cellular partners: 1) heterotrimeric G-proteins (Gαβγ), 2) β-arrestins, and 3) G-protein-coupled receptor kinases (GRKs) (4). A key evolutionary feature of GPCRs lies in their dynamic nature, which allows them to respond to different ligands and couple to distinct cellular partners (5,6). However, the dynamic nature of GPCRs makes it challenging to obtain structural information on intermediate activated states of signaling complexes, which are thermodynamically unstable but important for pharmacology.

In structural biology, various strategies have been developed to overcome limitations imposed by the intrinsic flexibility of GPCR signaling complexes. These include protein engineering to promote crystal lattice formation for x-ray crystallography, as well as development of protein binders that serve as physical anchors to facilitate crystal formation or to assist with single-particle analysis in cryoelectron microscopy (cryo-EM) data processing (7). In some cases, protein binders artificially stabilize transient conformational states that would otherwise be difficult to capture. Expanding the repertoire of binders that target GPCR signaling complexes offers new opportunities for both structural and functional studies.

In this study, we report the biochemical and structural characterization of two Fab fragments, Fab79 and Fab13, generated from mice immunized with the bovine rhodopsin (Rho)-Gαiβγ complex. Fab79 binds the α-helical domain (AHD) of Gαi, while Fab13 binds to the Gβ subunit. These two Fab fragments exhibit biochemical and structural impacts that are distinct from Gs-specific nanobody 35 (Nb35) (8) and Nb37 (9), and Gi-specific Fab16/scFv16 (10,11), which are currently widely used in the GPCR field. We propose that our two new antibody Fab fragments represent valuable additions to the existing repertoire of protein binders available to the community for studying the functional mechanisms underlying diverse signaling events.

## Material and methods

### Identification of binders for Fab13, Fab16, and Fab79

Fab13, Fab16, Fab79, Gαi, Gβγ, and Gαiβγ heterotrimers were prepared as described (11). Samples containing 0.48 mg/mL Fab were mixed separately with Gαi (0.19 mg/mL), Gβγ (0.19 mg/mL), or Gαiβγ heterotrimer (0.39 mg/mL) in a final volume of 100 *μ*L for analytical size-exclusion chromatography (SEC). This corresponds to a Fab/binder molar ratio of 2:1. Samples were incubated on ice for >1 h. A volume of 80 *μ*L was injected onto a self-packed Superdex 200 10/200 column equilibrated with 20 mM HEPES (pH 7.5), 150 mM NaCl, and 0.01% LMNG.

### Fab13, Fab16, and Fab79 for Rho-Gαiβγ integrity

The Rho-Gαiβγ complex purified in 0.01% LMNG was prepared as described (11). Samples containing Rho-Gαiβγ (0.58 mg/mL), with or without Fab (0.48 mg/mL), were prepared to 100 *μ*L and incubated on ice for >1 h. To induce complex dissociation, 0.1 mM GTPγS was supplemented to the samples and incubated for >1 h before SEC analysis. SEC runs were performed in the same manner as described above.

### Binding of Fab16 and Fab79 to Rho-Gαiβγ

Rho-Gαiβγ and Rho-Gαiβγ-Fab16 were prepared as described (11). Samples of Rho-Gαiβγ-Fab16 (1.9 mg/mL) alone or mixed with Fab79 (0.8 mg/mL) were prepared. In parallel, Rho-Gαiβγ (1.3 mg/mL) was mixed with both Fab16 (0.8 mg/mL) and Fab79 (0.8 mg/mL) and incubated for >1 h. The Rho-Gαiβγ/Fab16/Fab79 mixture was then supplemented with 0.1 mM GTPγS and incubated for an additional hour before SEC analysis using a Superdex 200 Increase 10/300 column equilibrated with 20 mM HEPES (pH 7.5), 150 mM NaCl, and 0.01% LMNG.

### Complex formation

Rhodopsin purification, complex formation with Gαiβγ heterotrimer, and Fab preparation were carried out as described (11). The single-chain antibody scFv16 was prepared as described (10). For cryo-EM, the Rho-Gαiβγ-scFv16-Fab79 complex was assembled by mixing Rho-Gαiβγ, scFv16 and Fab79 at a molar ratio of 1:1.4:1.4 in the presence of 0.02% LMNG and incubated overnight on ice. The mixture was concentrated using a 100-kDa MWCO concentrator before injection onto a Superdex 200 Increase 10/300 column equilibrated in 20 mM (pH 7.5) HEPES and 100 mM NaCl. The elution peak corresponding to Rho-Gαiβγ-scFv16-Fab79 was collected. The same procedure was used for preparing Rho-Gαiβγ-Fab13. Fab13 and Fab79 sequences are listed in Table S1.

### Rho-Gαiβγ-scFv16-Fab79 cryo-EM data collection and processing

Purified samples were plunge-frozen using a Vitrobot Mark IV machine (Thermo Fisher Scientific, Waltham, Massachusetts, USA) operated at 4°C and 100% humidity. A drop of 3.5 *μ*L sample (0.7 mg/mL) was applied onto a glow-discharged Quantifoil 1.2/1.3 copper grid and blotted for 3 s before plunge-freezing in liquid ethane. Data were collected using a Titan Krios electron microscope (Thermo Fisher Scientific) at the Scientific Center for Optical and Electron Microscopy (ScopeM), ETH Zürich (Zürich, Switzerland). Movies (40 frames) were acquired with a K2 camera (Gatan, Pleasanton, California, USA) at a nominal 1,650,00× magnification (0.854 Å/pixel) in counting mode with a final dose of 60 e^−^/Å^2^ and a defocus range of −0.8 to −2.0 *μ*m. A small data set of 1139 movies was collected to assess sample quality, followed by a larger data set comprising 14,027 movies. Both data sets were subjected to motion correction and patch contrast transfer function (CTF) estimation using cryoSPARC v.2.13 (12). Micrographs with CTF fit worse than 6 Å were excluded. Particle picking of the large data set was initiated by template picking, where the template was generated by blob picking from 2000 micrographs followed by 2D classification and class selection from the initial data set, leading to 7,026,029 particles picked. After a few runs of 2D classification and class selection, 771,361 particles from the 2D classes clearly showing Fab79 features were subjected to 3D heterogeneous refinement (5 classes). Selected particles from the small (50,702 particles) and the large (647,000 particles) data sets were exported to RELION 3.1 (13). Particles were subjected to 3D classification with or without solvent mask, resulting in maps clearly showing seven transmembrane helices. After the final 3D classification run, two classes (class 2, 98,000 particles; class 5, 118,000 particles) were refined to 5.2 and 5.9 Å, respectively (Fig. S2). The processing workflow is shown in Fig. S1.

### Rho-Gαiβγ-Fab13 cryo-EM data collection and processing

A 3 *μ*L volume of Rho-Gαiβγ-Fab13 (4 mg/mL) was applied to a glow-discharged Quantifoil EM copper grid (200 mesh, R1.2/1.3) and plunge-frozen in liquid ethane using a Vitrobot Mark IV machine. Data were collected on a Titan Krios (300 kV) equipped with a Gatan K3 camera and a BioQuantum energy filter. Movies were acquired at 1,300,00× nominal magnification in superresolution mode with the defocus range from −0.8 to −2.0 *μ*m. Each movie had 50 frames with a total dose of 66 e^−^/Å^2^.

The processing workflow is summarized in Fig. S5. A total of 25,818 movies were imported into cryoSPARC v.3.3.1 (12), motion-corrected, binned 2× (final pixel size 1.3 Å), and CTF-fitted using Patch CTF. After excluding micrographs that showed a CTF fit resolution worse than 6 Å, 23,221 micrographs were subject to Gaussian blob picking, yielding 3,752,145 picked particles. Following 20 rounds of 2D classification and class selection, 686,570 particles were selected and used for ab initio 3D reconstruction followed by heterogeneous refinement with 2 classes. One class showing better density of rhodopsin was used for nonuniform refinement using 587,113 particles, which were further cleaned from the previous particle set. Applying a mask to exclude the flexible regions (Gα-AHD and the distal part of Fab13) resulted in a density map at 3.7 Å resolution. Further heterogeneous refinement and nonuniform refinement with the selected 587,113 particles yielded a final map at 3.21 Å resolution, which was used for model building.

### Rho-Gαiβγ-scFv16-Fab79 model building and structure refinement

Initial models for rhodopsin (PDB: 6FUF) (14), the Gαi Ras domain and Gβ1γ1 (PDB: 6QNO) (11), and scFv16 (PDB: 6CRK) (10) were used. Gαi-AHD and Fab79 were modeled using AlphaFold (15). All components were docked into the 3D maps using UCSF Chimera (16), and real-space refinement was performed with Phenix (v.1.20.1-4487) (17), using PDB: 6QNO, 6FUF, and 6CRK as reference models. This procedure was performed for both conformations. Statistics are provided in Tables S2 and S3.

### Rho-Gαiβγ-Fab13 model building and structure refinement

Initial model of rhodopsin was taken from PDB: 5EN0 (18) and manually corrected for mutations I94T and M257Y using COOT (19). Gαi αN and α5 helices were taken from PDB: 6CMO (20), the rest of the Ras domain from PDB: 6CRK (10), and Gβ1γ1 from PDB: 1GOT (21). Fab13 was modeled using AlphaFold (15). Rigid-body docking was performed in UCSF Chimera (16), and real-space refinement was performed using Phenix (v.1.20.1-4487) (17), with PDB: 6FUF as reference model. Statistics are listed in Table S4.

### Figure preparation

Figures were prepared using UCSF ChimeraX (22) and PyMOL (The PyMOL Molecular Graphics System, v.2.0, Schrödinger, New York, New York, USA).

## Results

**Figure 1:**
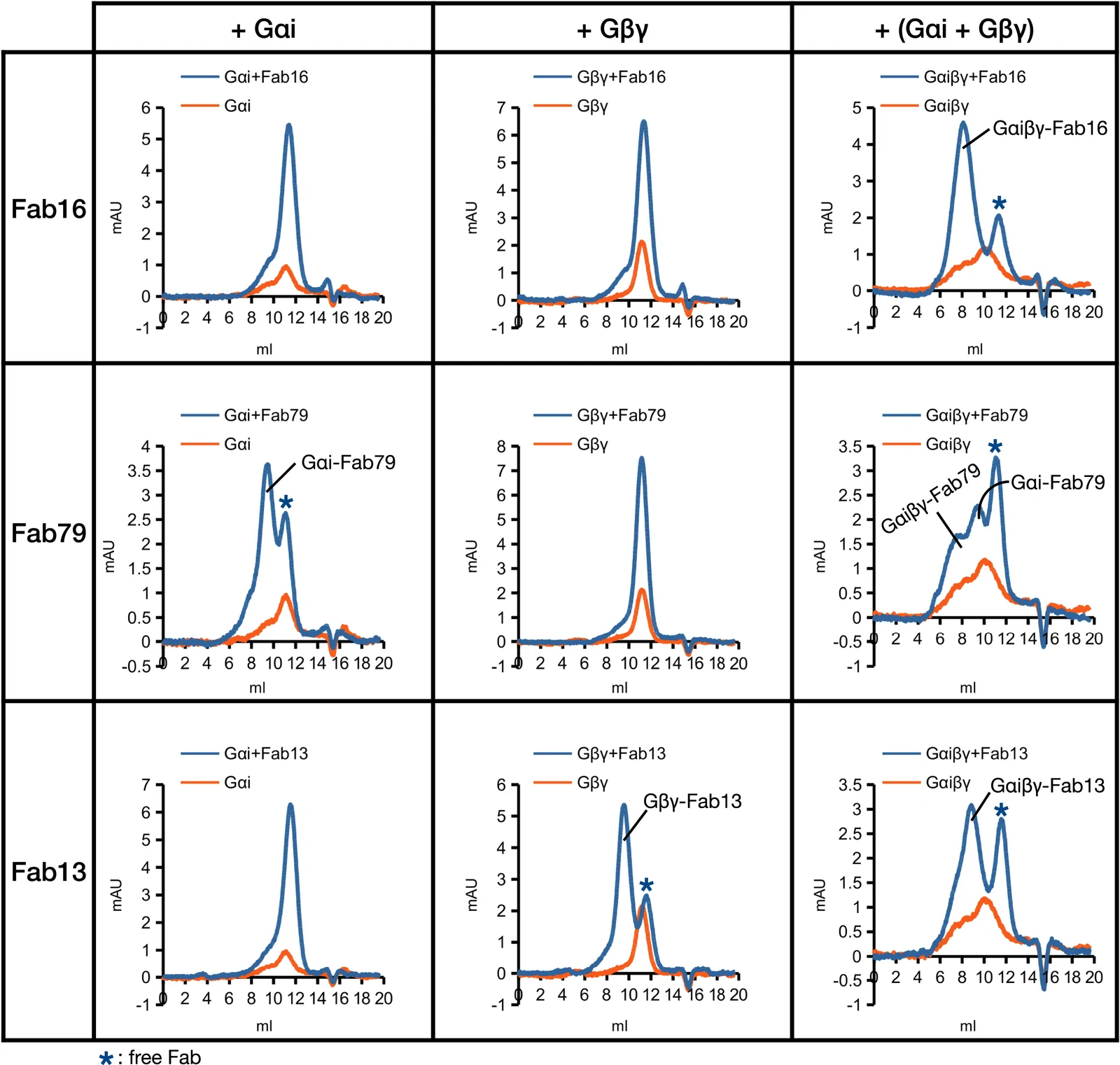
Fab binding to G-protein accessed by analytical SEC. SEC profiles of Fab16, Fab79, and Fab13 mixed with Gαi (*left column*), Gβγ (*middle column*), or the Gαiβγ heterotrimer (*right column*) under nucleotide-free condition. Peaks marked with blue asterisks indicate unbound Fab fragments.

Along with the discovery of Fab16/scFv16, two Fab fragments, Fab79 and Fab13, were identified in the same experiment using the monoclonal antibody generation approach in mice immunized with the purified Rho-Gαiβγ complex (10). To determine which component of Rho-Gαiβγ the antibodies bind to, purified Fabs were incubated with human Gαi1 (Gαi), bovine Gβ1γ1 (Gβγ), and the G-protein heterotrimer formed by Gαi and Gβγ (Gαiβγ). These Fab-G-protein mixtures were characterized by analytical SEC under nucleotide-free conditions. The SEC profiles reveal that Fab16 binds to the Gαiβγ heterotrimer, while Fab79 binds to Gαi, and Fab13 to Gβγ, respectively (Fig. 1).

### Fab79 and Fab16 stabilize the Rho-Gαiβγ complex in the presence of GTPγS

**Figure 2:**
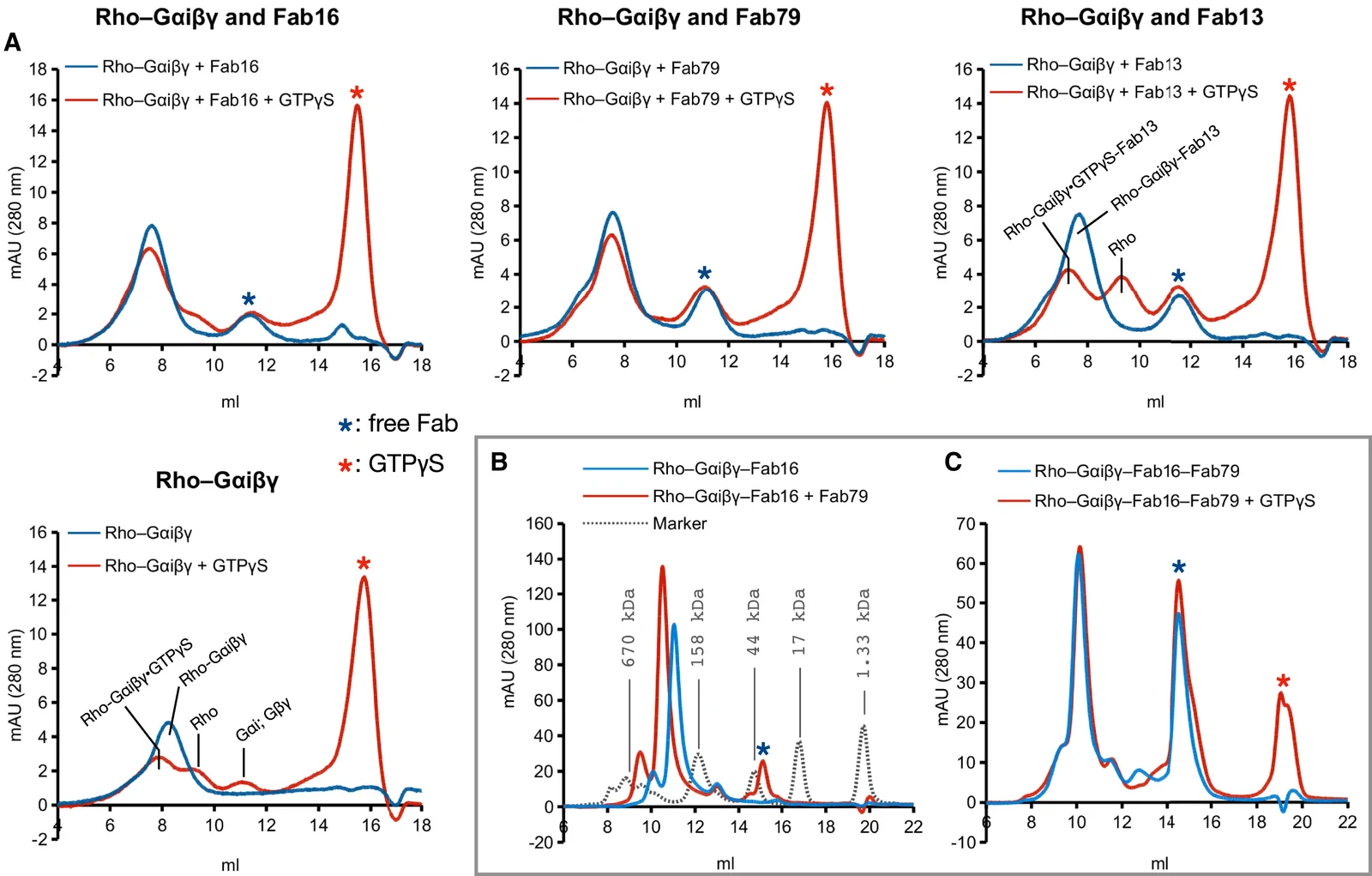
Analytical SEC of the Rho-Gαiβγ complex mixed with Fabs and GTPγS. (*A*) SEC profiles of Rho-Gαiβγ alone (*lower left*) or mixed with Fab16 (*upper left*), Fab79 (*upper middle*), or Fab13 (*upper right*), shown in blue curves. GTPγS was added to each sample to assess whether Fab binding prevents GTPγS-induced dissociation of the complex (*red curves*). Peaks corresponding to free Fab and GTPγS are marked with blue and red asterisks, respectively. (*B*) SEC profiles demonstrate that Fab79 can co-bind with Fab16, forming a stable Rho-Gαiβγ-Fab16-Fab79 complex. (*C*) The Rho-Gαiβγ-Fab16-Fab79 complex remains intact in the presence of GTPγS. SEC curves in (*A*) were obtained using a self-packed Superdex 200 10/200 column, whereas those in (*B*) and (*C*) were collected using a Superdex 200 Increase 10/300 column.

The nonhydrolyzable guanosine triphosphate (GTP) analog, GTPγS, is commonly used to trigger dissociation of GPCR-G-protein complexes (23,24). To test whether the purified Fab fragments can stabilize the Rho-Gαiβγ complex under these conditions, we incubated purified Rho-Gαiβγ with individual Fabs, followed by the addition of GTPγS. The integrity of the complex was then assessed by analytical SEC. Both Fab79 and Fab16 effectively preserved the complex and prevented dissociation of Gαiβγ from rhodopsin. In contrast, Fab13 did not stabilize the complex in the presence of GTPγS (Fig. 2 *A*). Upon complex dissociation, peaks corresponding to rhodopsin and individual G-protein subunits appeared at lower molecular weights. An additional peak eluting at an earlier retention volume was observed for Rho-Gαiβγ and Rho-Gαiβγ-Fab13 in the presence of GTPγS. We interpret this peak not as aggregation but as a distinct complex bound to GTPγS, where closure of Gα alters the overall shape and hydrodynamic properties, leading to earlier elution. Peak assignments were validated using absorbance at 280 and 380 nm (Fig. S1).

### Fab79 binds the α-helical domain of Gαi and prevents AHD closure

Next, we asked whether the protective effect of Fab79 against GTPγS-induced dissociation is due to its ability to bind both Gαi and Gβ, as previously shown for Fab16 (11). To determine whether Fab16 and Fab79 recognize distinct epitopes on the Gαiβγ heterotrimer, we performed analytical SEC using preformed Rho-Gαiβγ-Fab16, followed by addition of Fab79. The resulting elution profile shows a shift to higher molecular weight upon adding Fab79, consistent with simultaneous binding and suggesting that Fab79 and Fab16 bind to different epitopes (Fig. 2 *B*). Moreover, the assembled Rho-Gαiβγ-Fab16-Fab79 complex remains intact in the presence of GTPγS, indicating resistance to GTPγS-induced dissociation (Fig. 2 *C*).

**Figure 3:**
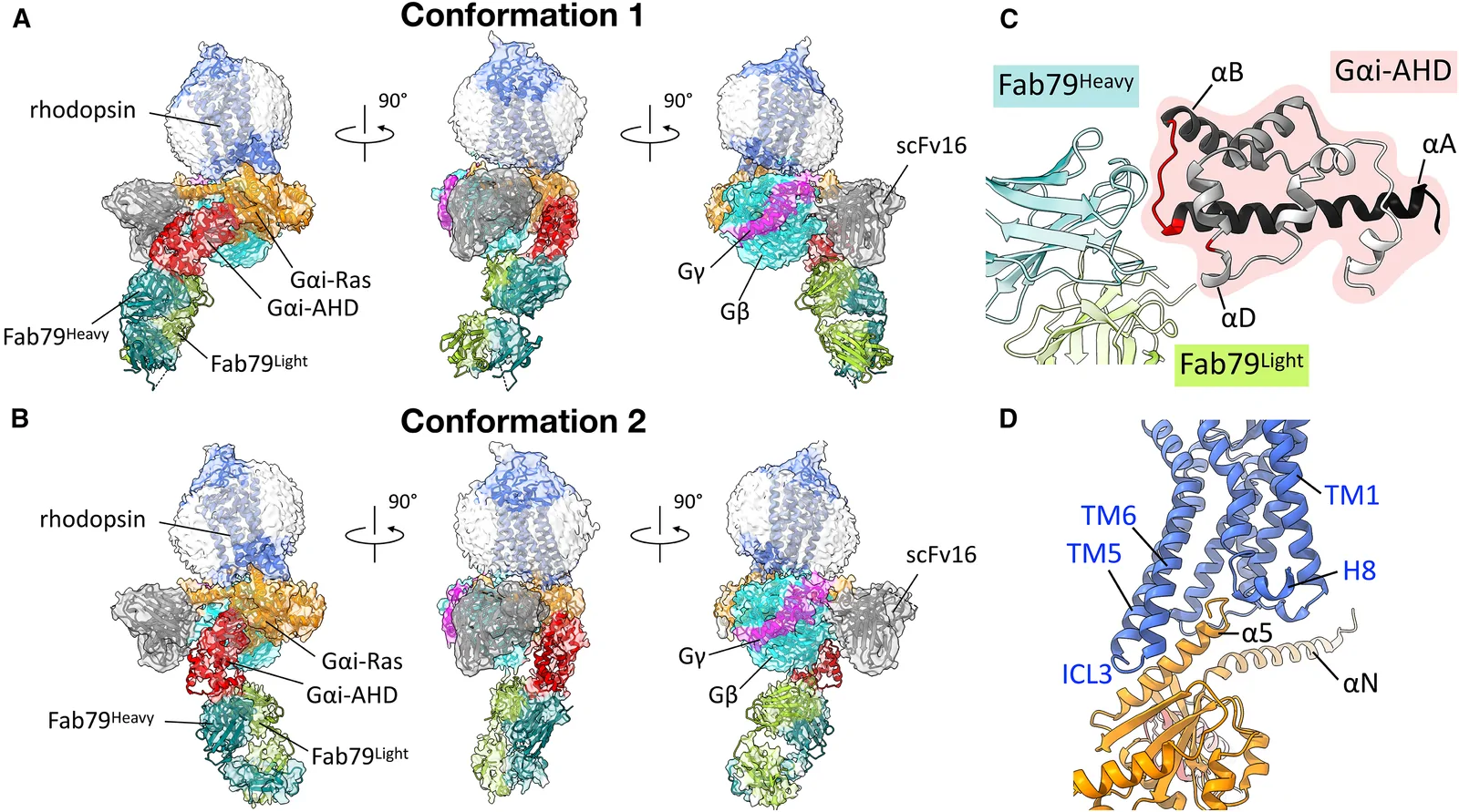
Structure and conformational flexibility of the Rho-Gαiβγ-scFv16-Fab79 complex. (*A*) Conformation 1 of Rho-Gαiβγ-scFv16-Fab79. (*B*) Conformation 2 of Rho-Gαiβγ-scFv16-Fab79. (*C*) Detailed view of the interface between Gαi-AHD and Fab79. Regions in Gαi-AHD that contact Fab79 are marked in red. (*D*) Binding interface between rhodopsin and the Gαi Ras domain. In (*A*), (*B*), and (*D*), protein components are colored as follows: rhodopsin (*blue*), Gαi-Ras (*orange*), Gαi-AHD (*red*), Gβ (*cyan*), Gγ (*magenta*), Fab79 light chain (*grass green*), Fab79 heavy chain (*teal*), and scFv16 (*gray*). The density representing detergent micelle is shown in white.

To visualize the binding epitope of Fab79, we performed cryo-EM single-particle analysis on the purified Rho-Gαiβγ-scFv16-Fab79 complex (Fig. S2). Fab79 binds to the AHD of Gαi, and 3D classification reveals multiple orientations of Fab79, reflecting the known intrinsic flexibility of the AHD (9). This conformational heterogeneity limited the overall resolution of the density maps. Nevertheless, two classes exhibiting the most distinct AHD orientations were refined to overall resolutions of 5.2 and 5.9 Å, respectively (Figs. 3, *A* and *B* and S3). Although the resolution of the density map is moderate, the reconstructions clearly show that Fab79 binds to the AHD primarily at the loop between αA and αB helices, and partially at the interface formed by the αA and αD helices (Figs. 3 *C* and S4 *A*). In both structures, scFv16 binds rigidly at the Gαi αN helix and the Gβ subunit as expected. Rhodopsin adopts the hallmarks of an activated receptor, as evidenced by the outward movement of transmembrane helix 5 (TM5) and TM6, and the insertion of the C-terminal α5 helix of the Gαi subunit into the cytoplasmic cleft of the receptor (Fig. 3 *D*). To quantity the conformational differences, we aligned both modeled structures to rhodopsin and calculated root mean-square deviation (RMSD) for carbon α atoms (Cα) across each component. Both conformations align well for rhodopsin (average Cα RMSD 1.34 Å), the Gαi Ras domain (2.30 Å), Gβ (1.27 Å), Gγ (1.57 Å), and scFv16 (1.81 Å). In contrast, Gαi-AHD and Fab79 showed higher RMSD values due to their differing orientations: 13.11 Å for Gαi-AHD, 44.69 Å for the Fab79 heavy chain, and 40.53 Å for the light chain (Fig. S4 *B*). Despite this variability, the rhodopsin/Gαi-Ras domain core structure aligned well with previously reported complexes, including Rho-mini-Gαo (PDB: 6FUF) (14), Rho-Gαiβγ-Fab16 (PDB: 6QNO) (11), Rho-Gαiβγ-Fab_G50 (PDB: 6CMO) (20), and Rho-Gαtβγ with and without Nb35 (PDB: 6OY9, 6OYA) (25) (Fig. S5, *A* and *B*). The open positions of the AHD seen in both structures also agree with the published neurotensin receptor 1-Gi complex (PDB: 7L0Q, canonical state; 7L0S noncanonical state) and the jumping spider rhodopsin 1 (JSR1)-Gi/q complex (PDB: 9EPP, 9EPQ; both with AHD) (26). In those structures, Gαi shows a relatively similar open state (Fig. S5 *C*).

While the cryo-EM maps illustrate how Fab79 binds to Rho-Gαiβγ, they do not explain how Fab79 prevents complex dissociation in the presence of GTPγS. This is different from Fab16 and Fab_G50, which simultaneously bind Gα and Gβγ, thereby preventing the dissociation of the G-protein heterotrimer (10,20). Fab79 may sterically hinder the AHD from approaching the Ras domain to form the closed conformation, a structural transition thought to be required for unfolding the tip of the α5 helix and promoting complex dissociation (27,28). To evaluate this idea, we superimposed the structure of Fab79 bound to the AHD onto previously solved structures of Gα in its closed conformation (29,30). We found that Fab79 is located very close to the Ras domain in the closed conformation (Fig. S6 *A*). In these models, Fab79 is positioned in close proximity to the Ras domain, with clearly steric clashes at the loop between the β4 strand and the α3 helix (Fig. S6 *B*). Given the positions of AHD/Fab79 observed in our cryo-EM data, it is plausible that Fab79 reduces the likelihood of the AHD and Ras domain coming together to form the closed state.

### Fab13 binds Gβ at the N-terminus and blades 5–6, altering receptor-Gα interaction

**Figure 4:**
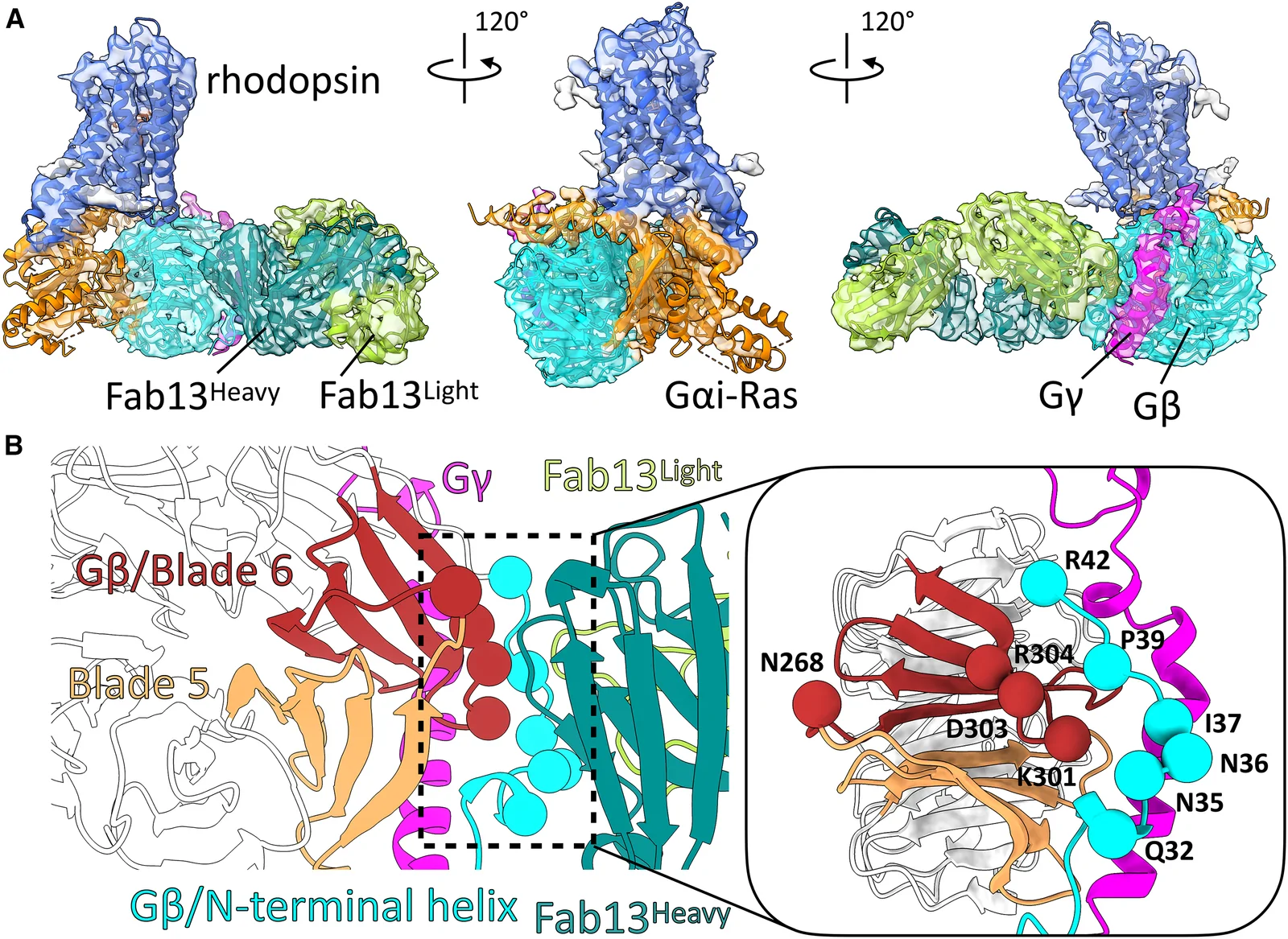
Cryo-EM structure of the Rho-Gαiβγ-Fab13 complex. (*A*) Cryo-EM density map and atomic model of Rho-Gαiβγ-Fab13. Protein components and their corresponding densities are colored as follows: rhodopsin (*blue*), Gαi (*orange*), Gβ (*cyan*), Gγ (*magenta*), Fab13 heavy chain (*teal*), and light chain (*grass green*). (*B*) Binding interface between Fab13 and Gβ. Gβ residues within 4 Å of Fab13 are displayed in spheres for their Cα. Blades 5 and 6 of the Gβ propeller are colored pale orange and maroon, respectively.

To investigate how Fab13 interacts with the Rho-Gαiβγ complex, we performed single-particle cryo-EM and obtained a density map at an overall resolution of 3.2 Å (Figs. S7 and S8). Although Fab13 binds the relatively rigid Gβ subunit, large regions of the density map, particularly the Gα subunit, exhibit poor resolution. This suggests that Fab13 may not stabilize the entire signaling complex as effectively as other binders, such as Fab16 (11) or Fab_G50 (20). Based on the map, we built a structural model (Fig. 4 *A*). Fab13 binds Gβ at an interface formed by the N-terminal helix (near Q32, N35-I37, P39, and R42; *cyan*), blade 6 (K301 and D303-R304; *maroon*), and the loop connecting blades 5 and 6 (N268; *maroon*) (Fig. 4 *B*). Detailed residue-residue contact is listed in Fig. S9 *A*.

**Figure 5:**
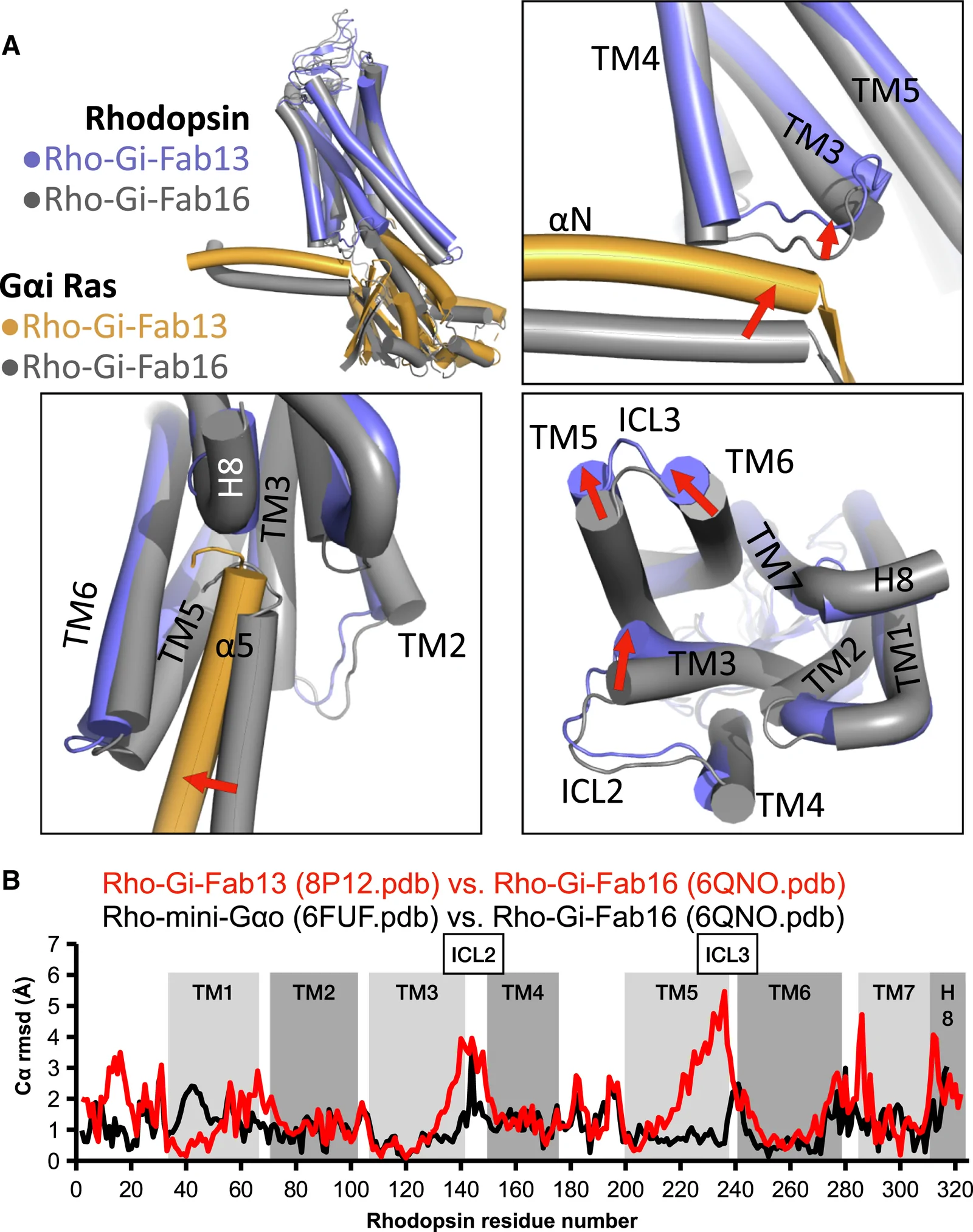
Structural comparison of Rho-Gαiβγ-Fab13 and Rho-Gαiβγ-Fab16. Rho-Gαiβγ-Fab13 and Rho-Gαiβγ-Fab16 (PDB: 6QNO) are superposed by aligning their rhodopsin components to the Cα of rhodopsin in Rho-mini-Gαo (PDB: 6FUF). (*A*) Overlay of Rho-Gαiβγ-Fab13 and Rho-Gαiβγ-Fab16 (*upper left panel*) and detailed comparisons at key interfaces of rhodopsin with the Gαi α5 helix (*lower left panel*), the Gαi αN helix (*upper right panel*), and the cytoplasmic side of rhodopsin (*lower right panel*). (*B*) RMSD of rhodopsin Cα between Rho-Gαiβγ-Fab13 and Rho-Gαiβγ-Fab16 (*red curve*), and between Rho-Gαiβγ-Fab16 and Rho-mini-Gαo (*black curve*).

Although the Gαi subunit was resolved at a lower local resolution, the density was sufficient to model the α5 helix backbone (Fig. S9 *B*). To investigate whether the coupling between rhodopsin and Gαi differs, we performed structural alignment between the Rho-Gαiβγ-Fab13 complex and other rhodopsin-G-protein complexes. This analysis revealed conformation changes at the rhodopsin/Gαi interface in the presence of Fab13, which were further validated by cross-examining the densities and model fit (Fig. S9 *C*). Notably, the cytoplasmic region of rhodopsin adopts a slightly more open conformation, with the TM5/intracellular loop 3 (ICL3)/TM6 and TM7/H8 displaced outward by ∼5 and ∼2 Å, respectively (Figs. 5 and S10). ICL2 also shifts ∼6 Å toward the membrane (Fig. S11 *A*). These structural shifts propagate through the Gα subunit. The α5 helix is shifted by ∼10° (Fig. S11 *B*), and its C-terminal hook inserts ∼4 Å deeper into the receptor core, likely due to the outward displacement of TM7/H8 (Fig. S10 *B*). Additionally, the αN helix of Gαi moves ∼9 Å toward the membrane, in parallel with the repositioning of ICL2 (Fig. S11 *A*). Taken together, these changes appear to originate from the repositioning of Gβ.

To assess Fab13-induced difference at the rhodopsin/Gβ and Gαi/Gβ interfaces, we compared the position of Gβ relative to rhodopsin by aligning several Rho-Gαiβγ complex structures to the rhodopsin Cα atoms in the Rho-Gαiβγ-Fab13 structure (Fig. S12 *A*). This analysis revealed that Gβ shifts toward the ICL1 region of rhodopsin upon Fab13 binding, with displacements of 9.1, 8.3, 8.6, and 12.5 Å compared with Rho-Gαiβγ-scFv16-Fab79 conformation 1, conformation 2, Rho-Gαiβγ-Fab16, and Rho-Gαiβγ-Fab_G50, respectively. Although Gβ itself does not undergo major conformational changes that would substantially alter its interface with Gαi (Fig. S12 *B*), this upward repositioning is, however, sufficient to modify the relative orientation of Gαi with respect to rhodopsin. These observations suggest that Fab13 modulates receptor-G-protein coupling primarily through repositioning of Gβ, rather than by inducing direct conformational changes within Gβ.

## Discussion

Antibody fragments and nanobodies are protein binders often used to facilitate structural studies of GPCRs and their signaling complexes (31,32,33,34). These binders have been developed to aid crystallization or to increase particle size, thereby facilitating alignment during EM data processing (7). Beyond antibody-based stabilization, fusion-protein strategies have been successfully applied to solve GPCR structures (33). This typically involves fusing soluble proteins such as BRIL and T4 lysozyme to receptor termini or ICL3 (35,36,37,38). However, such strategies are less compatible with structural studies of GPCR-G-protein complexes, as insertions near intracellular loops or the C-terminus can interfere with G-protein binding.

**Figure 6:**
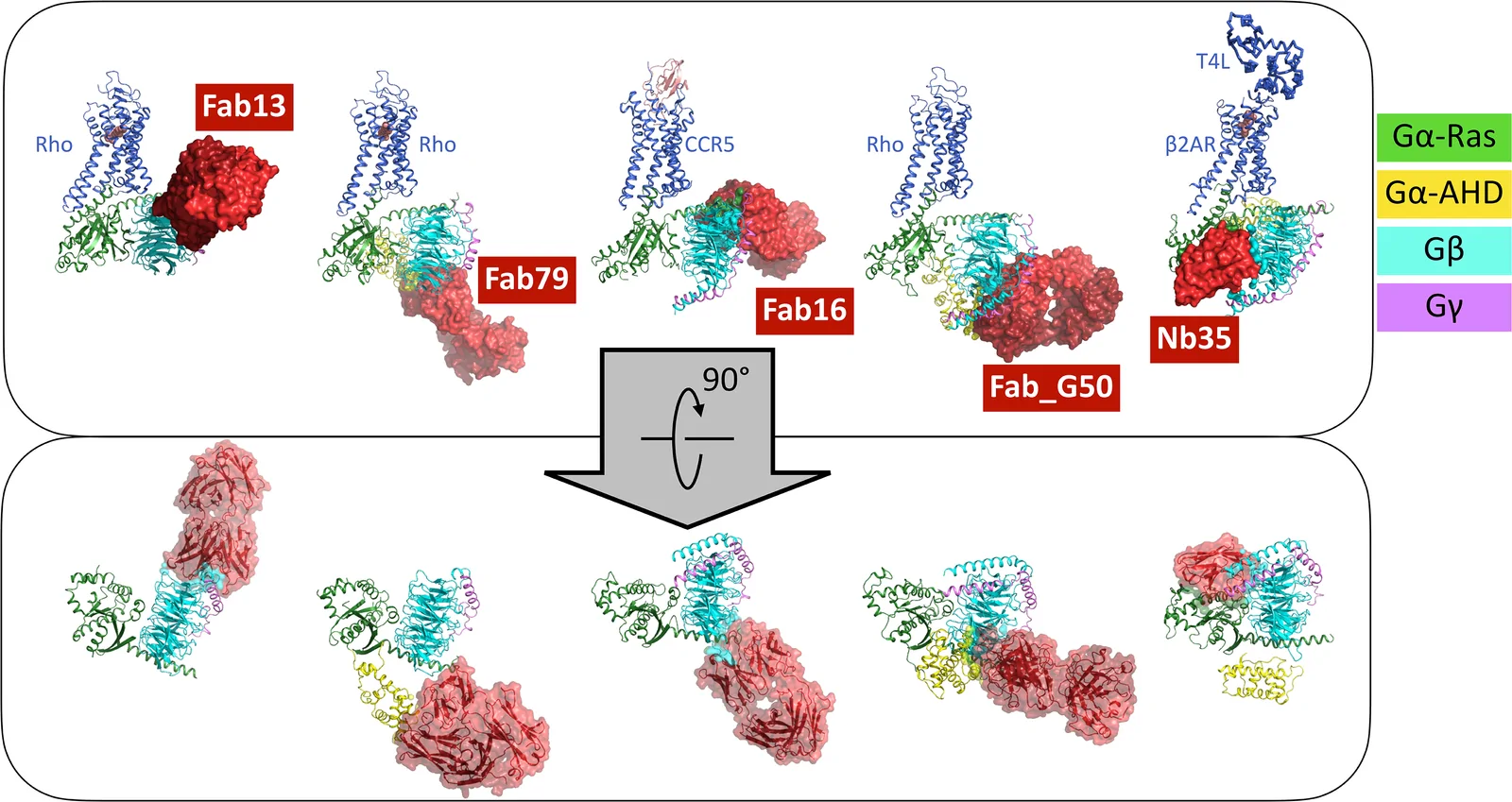
Protein binders used for structure determination of GPCR-G-protein complexes. GPCR-G-protein complexes with different binders are aligned with their receptors to the rhodopsin carbon α in Rho-Gαiβγ-Fab13. Fab13 (Rho-Gαiβγ; PDB: 8P12), Fab79 (Rho-Gαiβγ-scFv16 conformation 1; PDB: 8P13), Fab16 (CCR5-Gαiβγ; PDB: 7O7F), Fab_G50 (Rho-Gαiβγ; PDB: 6CMO), Nb35 (β2AR-Gαsβγ; PDB: 3SN6) are shown in red surfaces. Other protein components are color coded: receptors (*blue*), Gα-Ras (*green*), Gα-AHD (*yellow*), Gβ (*cyan*), Gγ (*magenta*), Fab or Nb (*red*). Structures in the upper panel are viewed through the membrane, and in the lower panel viewed from the cytoplasmic side.

For GPCRs, several binders have been shown to stabilize either inactive or active receptor states, such as nanobody 6 (Nb6) (34) as well as Nb80 (39) and Nb6B9 (40) for the β2 adrenergic receptor (β2AR), and Fab16/scFv16 that help visualize the C-terminal tail of rhodopsin (11) and M1 muscarinic acetylcholine receptor (41). For GPCR-G-protein complexes, binders may stabilize or destabilize certain conformations, and in some cases this can provide insights into structures that would otherwise be too transient to visualize under physiological conditions. A subset of antibody fragments and nanobodies have been selected specifically for their ability to prevent dissociation of signaling complexes, although these tend to be Gα-subtype specific. Examples include Nb35 for Gαs (8), Fab16/scFv16 (42), and Fab_G50 (20) for Gαi (Fig. 6; Table 1). These antibodies typically bind both Gα and Gβ subunits, stabilizing the heterotrimer and preventing its dissociation. This stabilization may result from inhibiting the separation of Gα and Gβγ subunits before receptor disengagement (43). As a result, Nb35 and Fab16/scFv16 are now widely used in cryo-EM studies of GPCR-G-protein signaling complexes.

**Table 1:** Summary of Fab13, Fab79, Fab16, Fab_G50, and Nb35 for their detailed epitopes and impacts in preventing GPCR-G-protein complexes from GTPγS-induced dissociation

	Fab13	Fab79	Fab16	Fab_G50	Nb35
Binders	Gβ	Gαi	Gαi and Gβ	Gαi and Gβ	Gαs and Gβ
Binding site on Gα	–	AHD: αA, αD helices, αaαb loop	N-terminus	AHD: αA helix + connecting loops	α2, α3, αG helices
Binding site on Gβ	N-terminus + blade 6	–	blades 1, 2	blades 3, 4	N-terminus + blades 4, 5, 6
Preventing GPCR-G-protein complex from nucleotide-induced dissociation	no	yes	yes	yes	yes

While many of these binders were initially developed to aid structural studies, the rapid progress of cryo-EM has made such stabilizers less critical. Instead, their functional roles in modulating receptor-G-protein interactions and conformational dynamics have become increasingly valuable. Despite the growing number of GPCR-G-protein complex structures solved by single-particle cryo-EM, most lack resolved density of the AHD of Gα due to its conformational flexibility with only a few exceptions (20,26,44). This flexibility is essential, as the AHD plays a central role in nucleotide exchange and complex dissociation, yet the dynamic behavior, ranging from receptor coupling, nucleotide exchange to receptor disengagement, remains poorly understood. Structural statistics suggest that Gαs subtype exhibits a uniformly flexible AHD, as observed in the time-resolved cryo-EM study of the β2AR-Gs complex upon addition of GTP (28). In contrast, the Gαi subtype appears to favor certain open poses of the AHD (PDB: 7L0Q, 7L0S, 9EPP, 9EPQ) (26,45), consistent with our Rho-Gαiβγ-scFv16-Fab79 structures, regardless of the presence of inhibitory antibodies. This indicates that the conformational dynamics of the AHD may differ across Gα subtypes. By preventing full closure of the Ras domain and the AHD, Fab79 is particularly suited for studying Gαi signaling dynamics, capturing states from precoupling to GPCR, to fully engaged complexes, and toward the nearly closed form of Gαi. Such an approach may also help to track the restructuring of the Gα C-terminus as it folds during the transition from precoupling to full receptor engagement, and to follow the dynamics of the fully coupled complex as it progresses toward GTP binding before dissociation.

Time-resolved structural biology provides a promising framework for probing GPCR signaling. Time-resolved x-ray crystallography has successfully captured light activation of rhodopsin, revealing receptor conformational changes and the isomerization of 11-*cis* to all-*trans* retinal (46). More recently, time-resolved cryo-EM has visualized the structural transitions of the β2AR-Gs complex (28) and the μ-opioid receptor-Gi complex (47) upon GTP binding, demonstrating that second-scale conformational dynamics of GPCR-G-protein complexes can now be resolved. As cryo-EM technologies continue to advance in temporal resolution and sample preparation strategies (48,49), new opportunities will arise to capture short-lived intermediates that are otherwise inaccessible. In this context, Fab79 is unique positioned to enrich specific conformational states of Gαi AHD and stabilize transient intermediates long enough for structural capture. Combining Fab79 with time-resolved cryo-EM could therefore shed light on how the Gαi AHD reorganizes during nucleotide exchange, how the receptor/Gαi interface remodels throughout the signaling cycle, and how these motions shape signaling output. A deeper understanding of these dynamic interaction will provide new insights into how conformation-dependent receptor/G-protein affinity encodes downstream signaling responses.

Fab79 and Fab13 reported in this study show different properties compared with previously characterized binders. Fab79 binds to the distal end of the Gαi-AHD, allowing us to capture two major open conformations of the AHD within the Rho-Gαiβγ complex. Notably, the AHD does not adopt a closed conformation in these structures, unlike in the crystal structures of isolated Gα or Gαβγ. Similar to Fab79, Nb37 binds the Gαs-AHD at the loop between αA and αB helices and prevents the β2AR-Gs complex from dissociation in the presence of GTPγS (8,9,50). The ability of Fab79 to prevent dissociation of the Gαβγ heterotrimer remains to be fully understood, but we speculate that it may result from interference with AHD closure, a conformational state required for receptor disengagement (28). A comparable mechanism has been proposed for Nb37, which was reported to stabilize the nucleotide-free state of Gαs (51). We therefore propose that Fab79 may similarly favor the nucleotide-free state of Gαi. This interpretation is also consistent with our SEC data, where the Rho-Gαiβγ·GTPγS peak was absent in the Fab79 data set. Nb37, since its discovery, has been repurposed for methodological applications such as single-molecule imaging (52) and as a biosensor to track GPCR signaling in endosomes (51,53,54). Similarly, we anticipate Fab79 or its engineered scFv/nanobody derivatives being adapted as new tools to probe Gi-mediated signaling, for example, by studying its inhibitory effect on adenyl cyclases and by tracking Gi-driven signaling from internalized GPCRs in endosomes.

Fab13, on the other hand, represents a valuable addition to the protein binder repertoire for GPCR-G-protein complexes, given the limited number of antibodies targeting Gβ or Gγ subtypes despite their key roles in signal transduction. Among the 16 Gα subtypes and 12 Gγ subtypes in humans, only 5 Gβ subtypes exist (55), making Gβ a relatively conserved target. The most commonly used Gβγ pairs in structural studies are Gβ1γ1 and Gβ1γ2. Fab13 binds to Gβ at an interface that includes conserved residues K301 and R304 (Fig. S13), suggesting that Fab13 may also bind other Gβ subtypes with minimal modification at the epitope binding site. Although Fab13 does not exhibit the same stabilization effect as Fab16 or Fab79, its binding appears to reposition Gβ and induce a slightly more open conformation in rhodopsin compared with previously reported structures (Figs. S10–S12). More studies are required to determine whether these conformational changes are truly induced by Fab13 or are specific to this case. The density map of Rho-Gαiβγ-Fab13 does not show high-resolution features that would allow for more detailed investigation. However, the density map clearly identifies the region of Gβ engaged by Fab13, providing valuable information to the community and enhancing the utility of this binder beyond its application in GPCR complexes.

Gβ is involved in signaling with other downstream partners such as GRKs, G-protein-coupled inwardly rectifying potassium channels (GIRKs), phosphoinositide 3-kinase γ (PI3Kγ), and guanylyl cyclases. Unlike Nb5, another Gβ binder that competes with Gα, phosducin, and GRK2 for Gβ binding (56), Fab13 binds a nonoverlapping epitope, suggesting that it could be used without disrupting canonical Gα-Gβγ or Gβγ-effector interactions or in conjunction to other binders such as Nb35 (8). Because of this property, Fab13 offers several opportunities: 1) it could be explored as a biochemical probe to isolate and stabilize Gβγ-effector complexes for structural or functional studies, 2) it may serve as a biosensor scaffold, engineered with fluorescence or conformational reporters to monitor spatiotemporal dynamics of Gβγ signaling in living cells, or 3) it may help dissect isoform-specific roles of Gβγ subtypes, given that its binding site is conserved across multiple Gβ variants. Developing Fab13 along these directions would significantly expand the experimental toolkit for studying Gβγ-mediated signaling, which is an area that remains comparatively underexplored in the GPCR field.

We noted the absence of clear retinal density in our cryo-EM maps. Upon comparison with previously reported rhodopsin-G-protein complex structures, we observed that retinal density is retained when samples are prepared under dim light (25), whereas our work including previously reported (11,14) and another study (20), all conducted under ambient light, show poor or no retinal density. We exclude low resolution as the primary reason for the absence of retinal because clear retinal density was observed in the bistable JSR1-Gαiβγ complex at a resolution of 4.9 Å (45). This suggests that rhodopsin bleaching, namely the transition from the meta II state (with all-*trans* 15-*anti* retinal) to meta III (with all-*trans* 15-*syn* retinal) and eventually to retinal release (57), occurs during binding to G-protein.

In conclusion, Fab79 provides a means to visualize and functionally modulate the dynamics of the Gα-AHD, while Fab13 represents an alternative binder for Gβ with broader applicability across G-protein signaling pathways. Although the effect of Fab13 on receptor conformation requires further investigation, its binding to Gβ supports its wider utility, especially for studying Gβγ signaling with downstream partners. Through biochemical and structural characterization of these novel binders, we provide the G-protein signaling field with new tools to explore their conformational regulation and signaling.

## Data and code availability

All data needed to evaluate the conclusions in this paper are present in the main text and/or the supporting material. Additional data related to this paper may be requested from the authors. The atomic coordinates and the EM density maps have been deposited to the Protein Data Bank (PDB) and the Electron Microscopy Data Bank (EMDB) under accession codes for Rho-Gαiβγ-Fab13 (PDB: 8P12, EMDB: EMD-17343), Rho-Gαiβγ-scFv16-Fab79 conformation 1 (PDB: 8P13, EMDB: EMD-17344), and conformation 2 (PDB: 8P15, EMDB: EMD-17345).

## Acknowledgments

We thank Shoji Maeda, Hugues Matile, and Roger Dawson for their pioneering work on generation of these antibodies. We are grateful to Guillaume Gotthard for helpful discussion during structural analysis, and to Miroslav Peterek at ScopeM, ETH Zürich, for his support during cryo-EM data collection. The work was supported by the 10.13039/501100001711Swiss National Science Foundation (SNSF) under grants 310030_153145, 310030B_173335, and 310030_192760, and by the 10.13039/501100000781European Research Council (ERC) under grant no. 951644. We also thank COST Action 18133 for European Research Network on Signal Transduction (ERNEST) for supporting networking and conference activities.

## Author contributions

F.P., O.T., J. Mühle, and C.J.-T. contributed in purification of rhodopsin and G-protein subunits. R.T. carried out antibody generation, selection, and sequencing. F.P., J. Mühle, and C.J.-T. prepared Fab fragments. F.P., O.T., and C.J.-T. conducted complex formation, purification, and cryo-EM grid preparation. F.P., O.T., and J. Marino performed EM data collection and processing. O.T. and C.J.-T. conducted model building and structural refinement. C.J.-T. conducted structural analysis on the atomic models. J. Marino, C.J.-T., and G.F.S.X. designed the experiment and wrote the manuscript.

## Declaration of interests

G.F.X.S. declares that he is a co-founder and scientific advisor of the companies leadXpro AG and InterAx Biotech AG.

## Supporting material

Document S1. Figures S1–S13 and Tables S1–S4

Document S2. Article plus supporting material
