# Supplementary material for: Tool antibody fragments reveal multiple conformations of the rhodopsin-Gi signaling complex
Source: Biophys J. 2025 Sep 29;125(10):2260–71. doi: 10.1016/j.bpj.2025.09.044 (PMC13351892; doi:10.1016/j.bpj.2025.09.044)
Supplement: Document S1. Figures S1–S13 and Tables S1–S4 [file mmc1.pdf]

Biophysical Journal, Volume *125*

Supplemental information

Tool antibody fragments reveal multiple conformations of the rhodopsin-Gi signaling complex

Filip Pamula, Oliver Tejero, Jonas Mühle, Ralf Thoma, Gebhard F.X. Schertler, Jacopo Marino, and Ching-Ju Tsai

Supplemental Information

Tool antibody fragments reveal multiple conformations of the rhodopsin-Gi signaling complex

Fillip Pamula[1,2,#], Oliver Tejero[1,2], Jonas Mühle[1], Ralf Thoma[3], Gebhard F. X. Schertler[1,2], Jacopo Marino[1], and Ching-Ju Tsai[1,*]

1) Laboratory of Biomolecular Research, Paul Scherrer Institute, Forschungsstrasse 111, 5232 Villigen PSI, Switzerland.
2) Department of Biology, ETH Zürich, Wolfgang-Pauli-Strasse 27, 8093 Zürich, Switzerland.
3) Pharma Research and Early Development (pRED), Roche Innovation Center Basel, F. Hoffmann-La Roche Ltd, Grenzacherstrasse 124, 4070 Basel, Switzerland

#) Current address: Department of Molecular Biology and Genetics, Aarhus University, 8000 Aarhus C, Denmark

*Correspondence for the manuscript:
Dr. Ching-Ju Tsai
Paul Scherrer Institute
CH-5232 Villigen PSI
Switzerland
Email: ching-ju.tsai@psi.ch
Telephone: +41 56 310 54 84

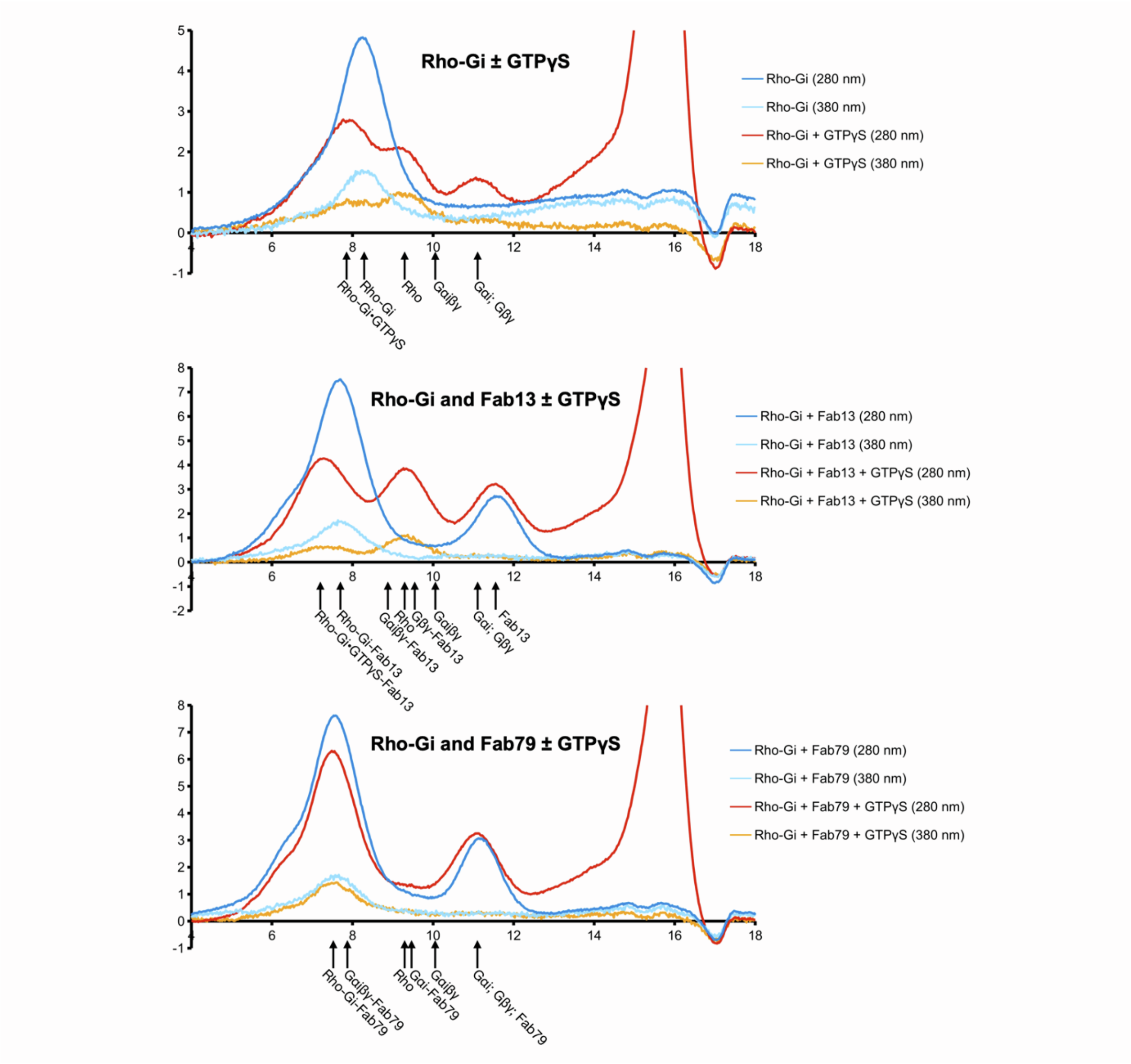

Figure S1. Analysis of SEC profiles of Rho-Gi-Fab complexes with and without GTPγS. The rhodopsin-Gαiβγ complex alone (top panel), with Fab13 (middle panel), or with Fab79 (bottom panel) were analyzed on a Superdex 200 10/200 column. Samples were supplemented with GTPγS for 1 hour prior to injection into the SEC column. UV absorption was monitored at 280 nm for protein and 380 nm for retinal. These runs correspond to those shown in Figure 2A. Possible protein assemblies are indicated at the retention volumes, based on the SEC data in Figure 1 and 2A.

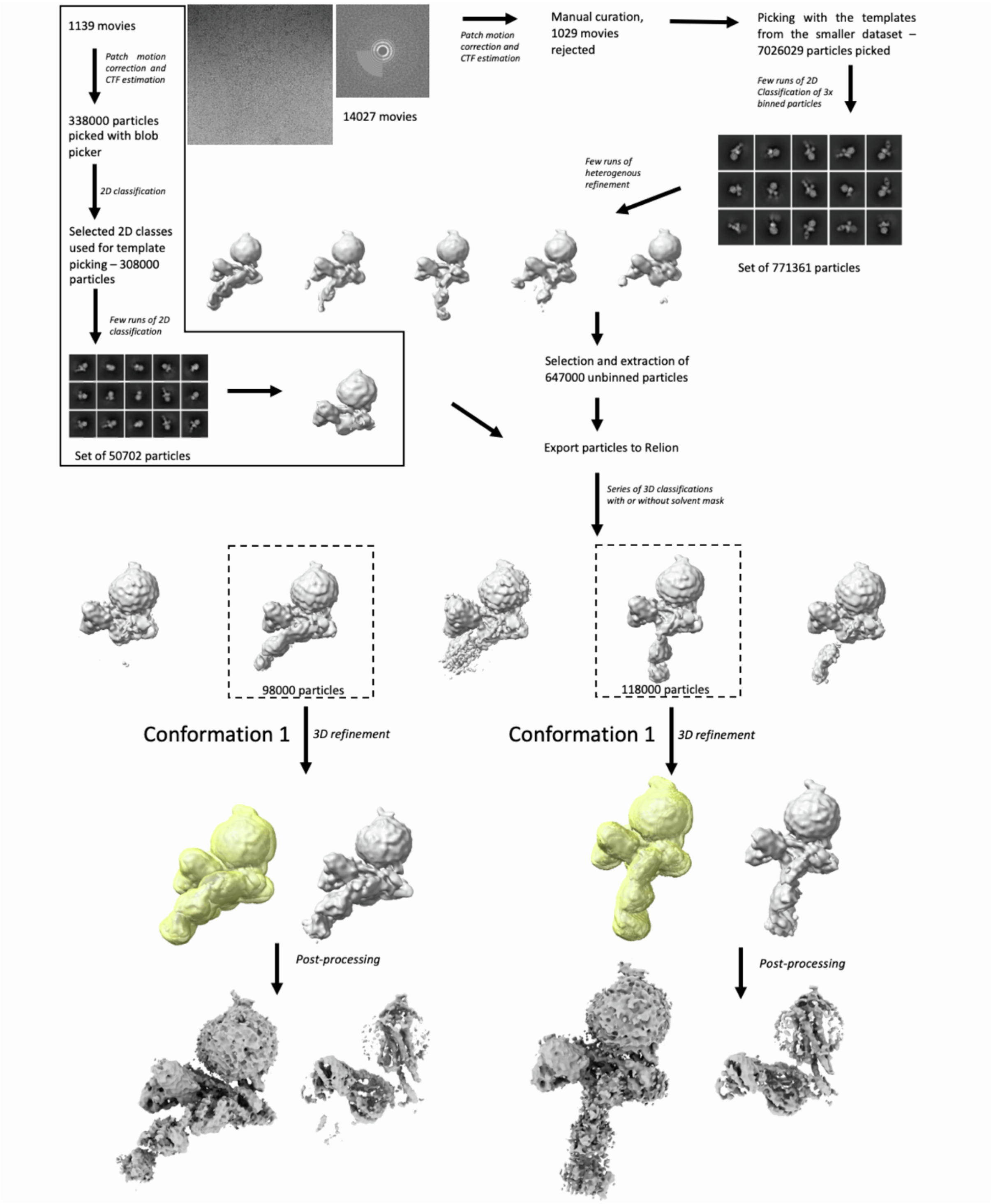

Figure S2. Cryo-EM data processing pipeline of Rho-Gαiβγ-scFv16-Fab79.

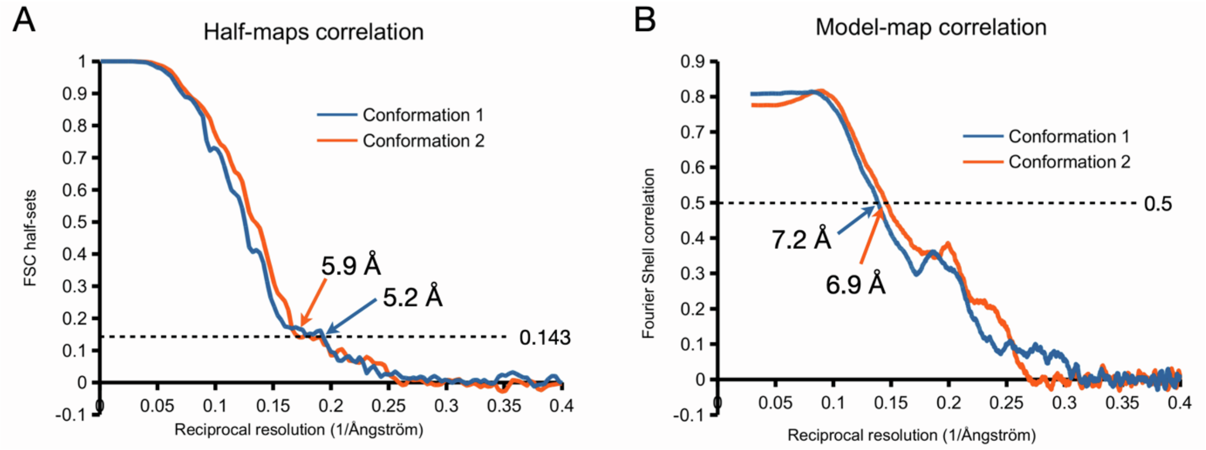

Figure S3. FSC curves of Rho-Gαiβγ-scFv16-Fab79. (A) Fourier shell correlation of the half-map datasets. **(B)** Fourier shell correlation of model and map.

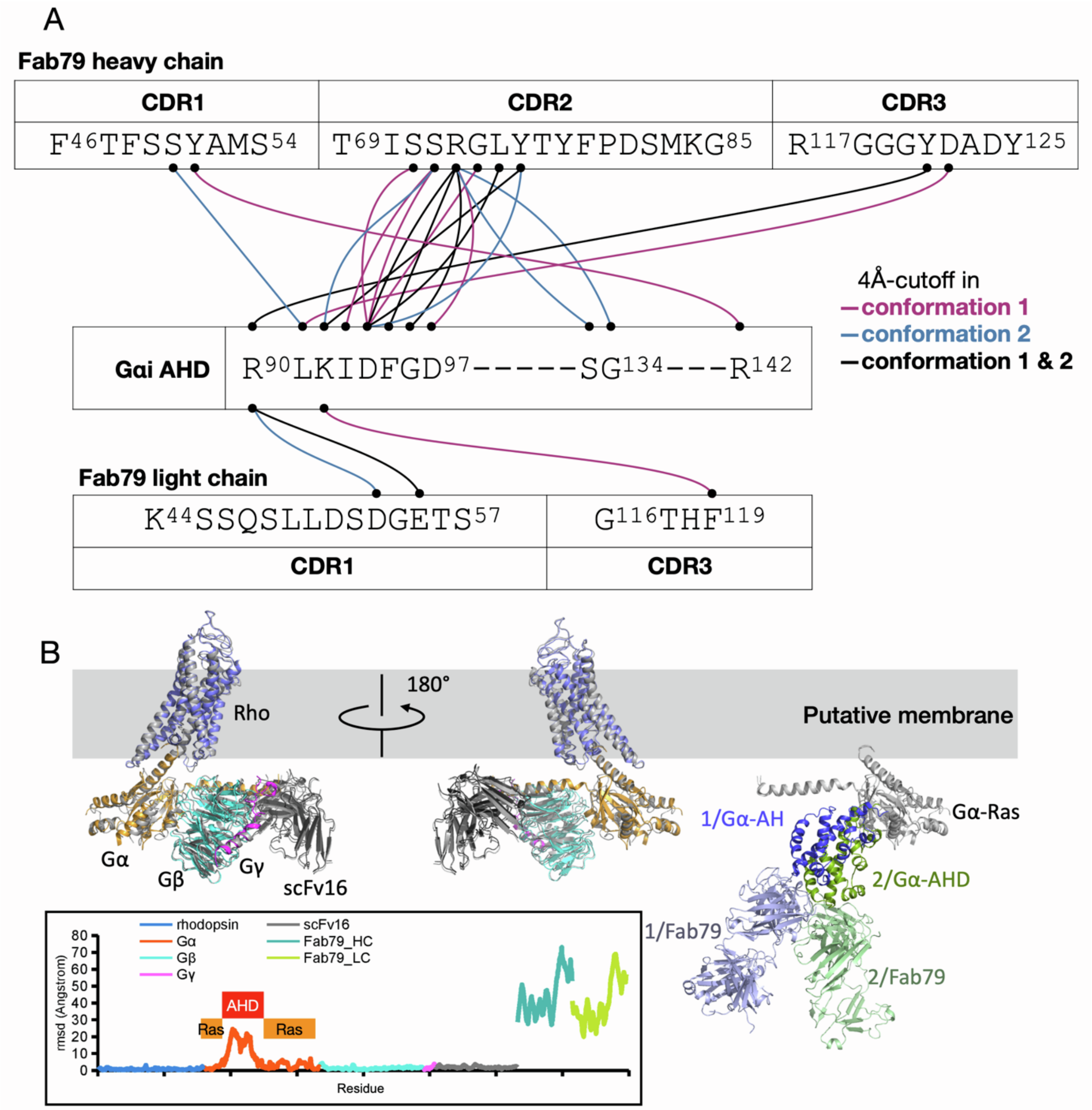

Figure S4. Structural insights into Fab79 binding to Rho-Gαiβγ. (A) Residue-residue contacts between Fab79 and the Gαi AHD, defined by a 4-Å cutoff, in both Rho-Gαiβγ-scFv16-Fab79 conformations. Contacts present in conformation 1, conformation 2, or in both conformations are colored in magenta, blue and black, respectively. Contact regions in Fab79 are confined to the complementarity-determining regions (CDRs). **(B)** The two conformations are aligned to the Cα atoms of rhodopsin. Left and middle overlays show the conformations without Gα-AHD and Fab79, and the right overlay displays Gα and Fab79, colored according to component labels. The inset shows the Cα r.m.s.d. between the two conformations.

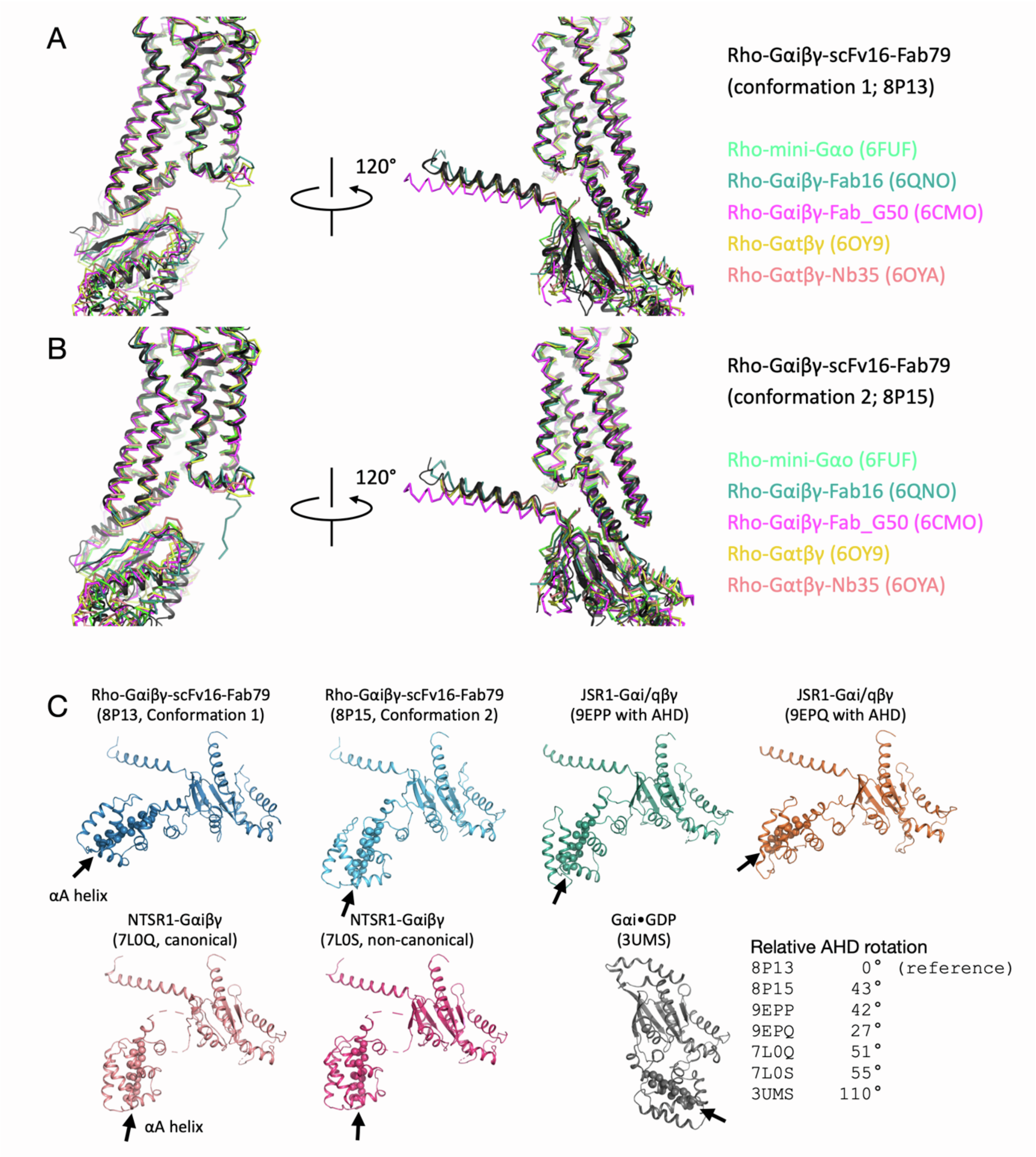

Figure S5. Structural comparison of Rho-Gαiβγ-scFv16-Fab79 with other GPCR-G protein complexes. (A, B) Comparison of conformation 1 **(A)** and conformation 2 **(B)** with previously reported active-state rhodopsin-G protein complexes. All structures are aligned to the rhodopsin Cα atoms in Rho-mini-Gαo (PDB 6FUF). Rho-Gαiβγ-scFv16-Fab79 is shown as cartoon, while the others are ribbons. **(C)** Comparison of Gαi AHD pose across Gi complexes of neurotensin 1 receptor (NTSR1) (PDB 7L0Q, 7L0S), Gi/Gq chimera complexes of JSR1 (PDB 9EPP, 9EPQ plus AHD), and GDP-bound Gαi (PDB 3UMS). All are aligned to the Cα atoms of the Gαi Ras domain (residues 1-51 and 183-354) of Rho-Gαiβγ-scFv16-Fab79 conformation 1 (PDB 8P13). Cα atoms of the AHD αA helix (residues 72-90) are shown as spheres to mark relative AHD positions. Rotation angles are measured through the αA helix, relative to Rho-Gαiβγ-scFv16-Fab79 conformation 1.

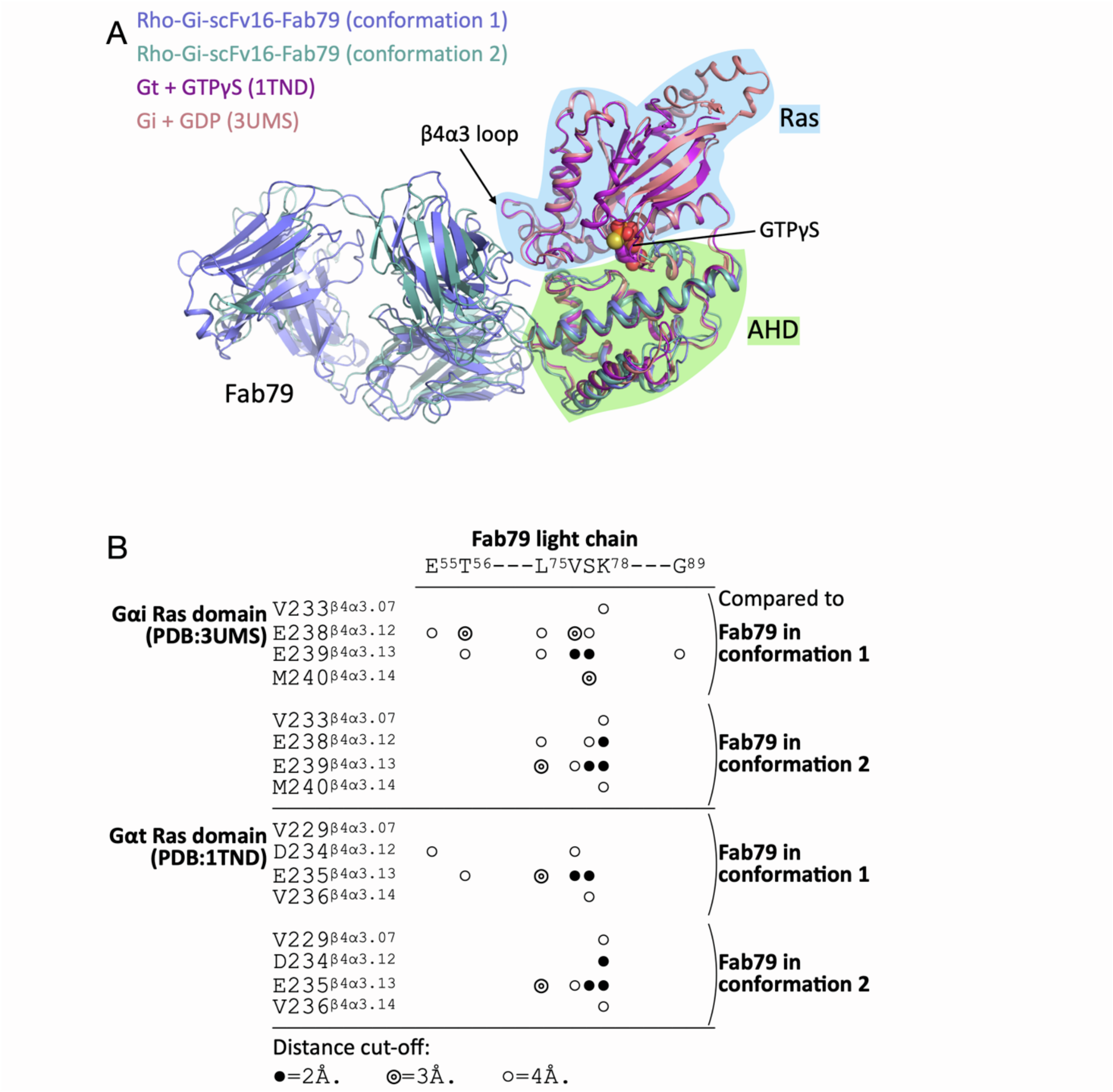

Figure S6. Structural comparison of the Gαi-AHD/Fab79 region with closed-state Gα proteins. **(A)** The closed-state Gα protein is represented by Gαt bound to GTPγS (PDB 1TND) and Gαi bound to GDP (PDB 3UMS). These structures, along with the two Rho-Gαiβγ-scFv16-Fab79 conformations, are aligned to Cα atoms of AHD. For the closed-state Gα proteins, Ras domain and AHD are shown. For Rho-Gαiβγ-scFv16-Fab79, Fab79 and AHD are shown. Ras domain is marked by a blue patch, and AHD by green. **(B)** Using the same alignment, clashes between residues of Fab79 and of nucleotide-bound Gα were identified ing a 2-4 Å cutoff for Fab79 in both conformations.

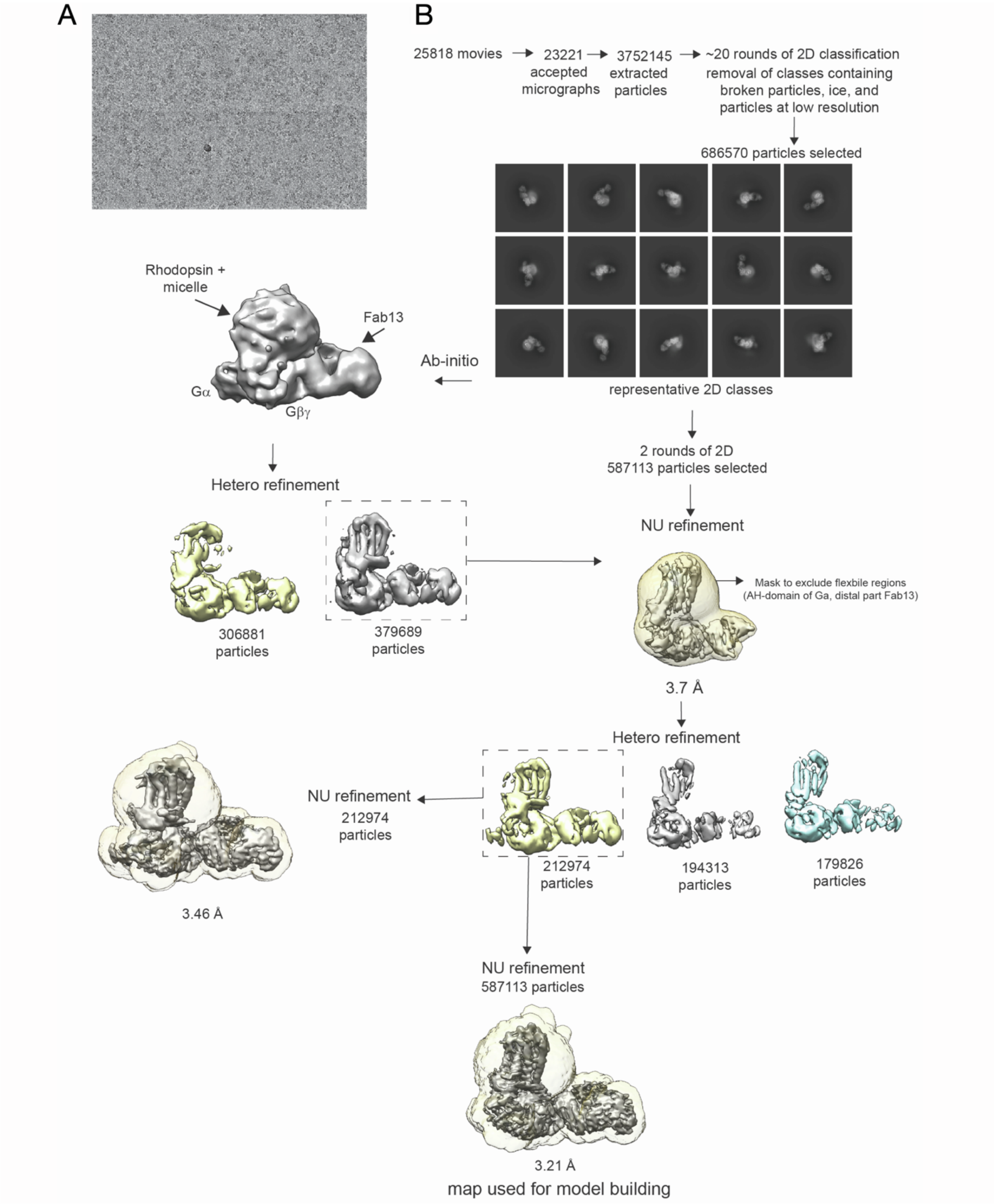

Figure S7. Cryo-EM data processing pipeline of Rho-Gαiβγ-Fab13.

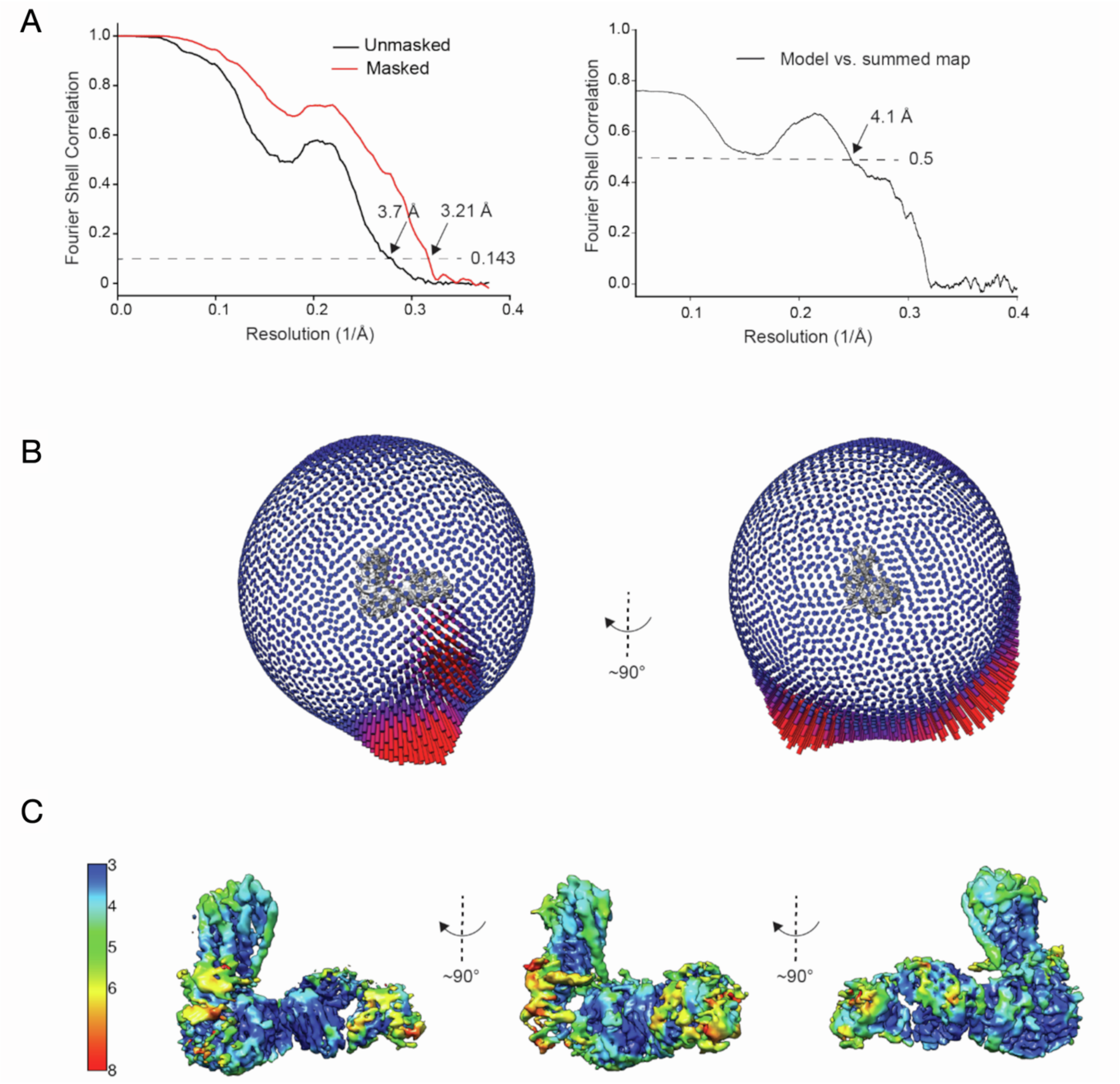

Figure S8. Resolution and particle distribution of Rho-Gαiβγ-Fab13. (A) Fourier shell correlation curves of the Rho-Gαiβγ-Fab13 map. **(B)** Angular distribution of the particles that contributed to the final density map. **(C)** Local resolution map calculated with BlocRes (1).

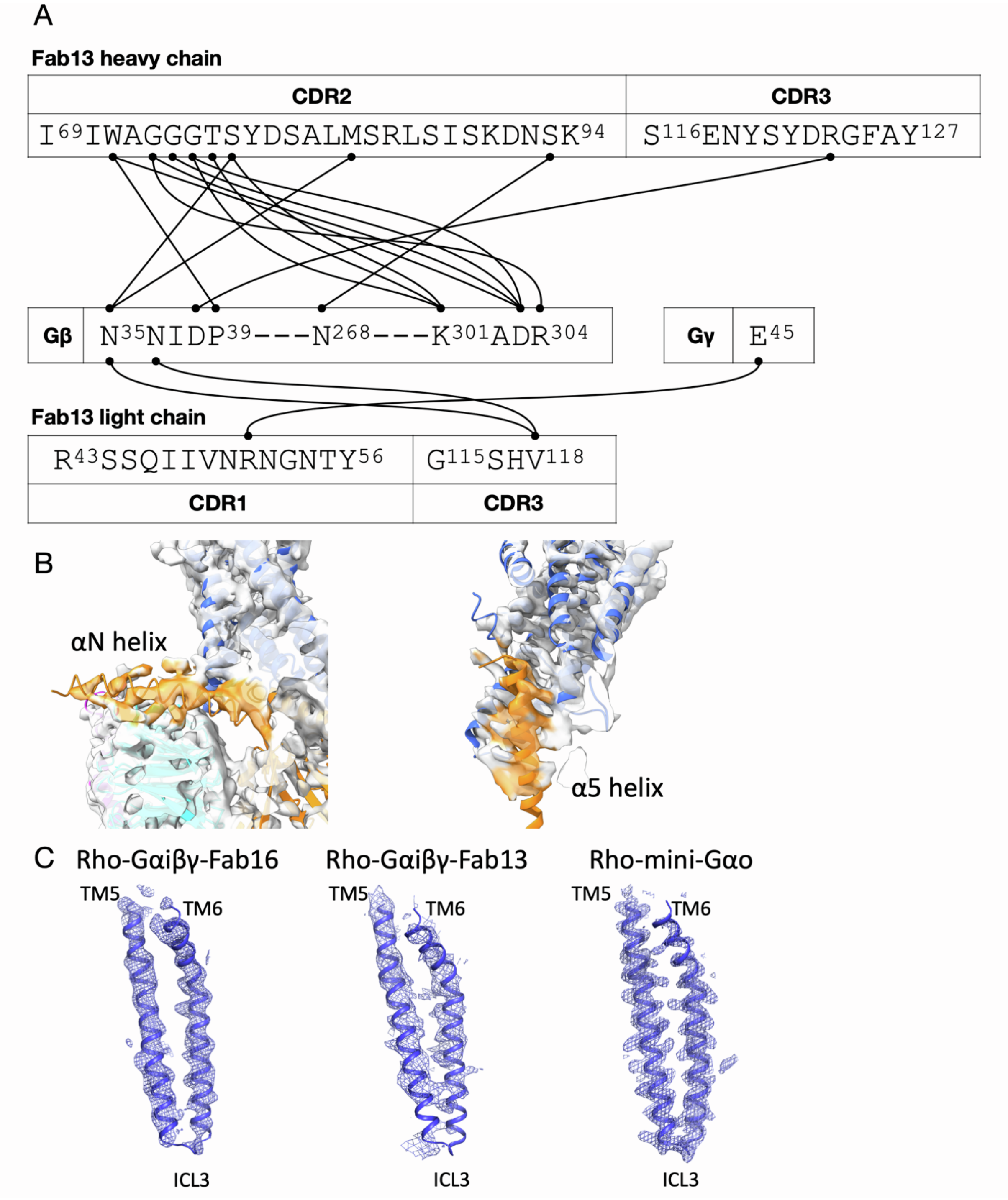

Figure S9. Structural details of Rho-Gαiβγ-Fab13. (A) Residue-residue contacts between Fab13 and Gβγ, defined by a 4 Å cutoff. Contacts are confined to the Fab13 CDRs. **(B)** Detailed view of the αN and α5 helices of Gαi in the Rho-Gαiβγ-Fab13 structure and map. **(C)** Comparison of the TM5/ICL3/TM6 region of rhodopsin in three complexes: Rho-Gαiβγ-Fab16 (left, PDB 6QNO), Rho-Gαiβγ-Fab13 (middle) and Rho-mini-Gαo (right, PDB 6FUF).

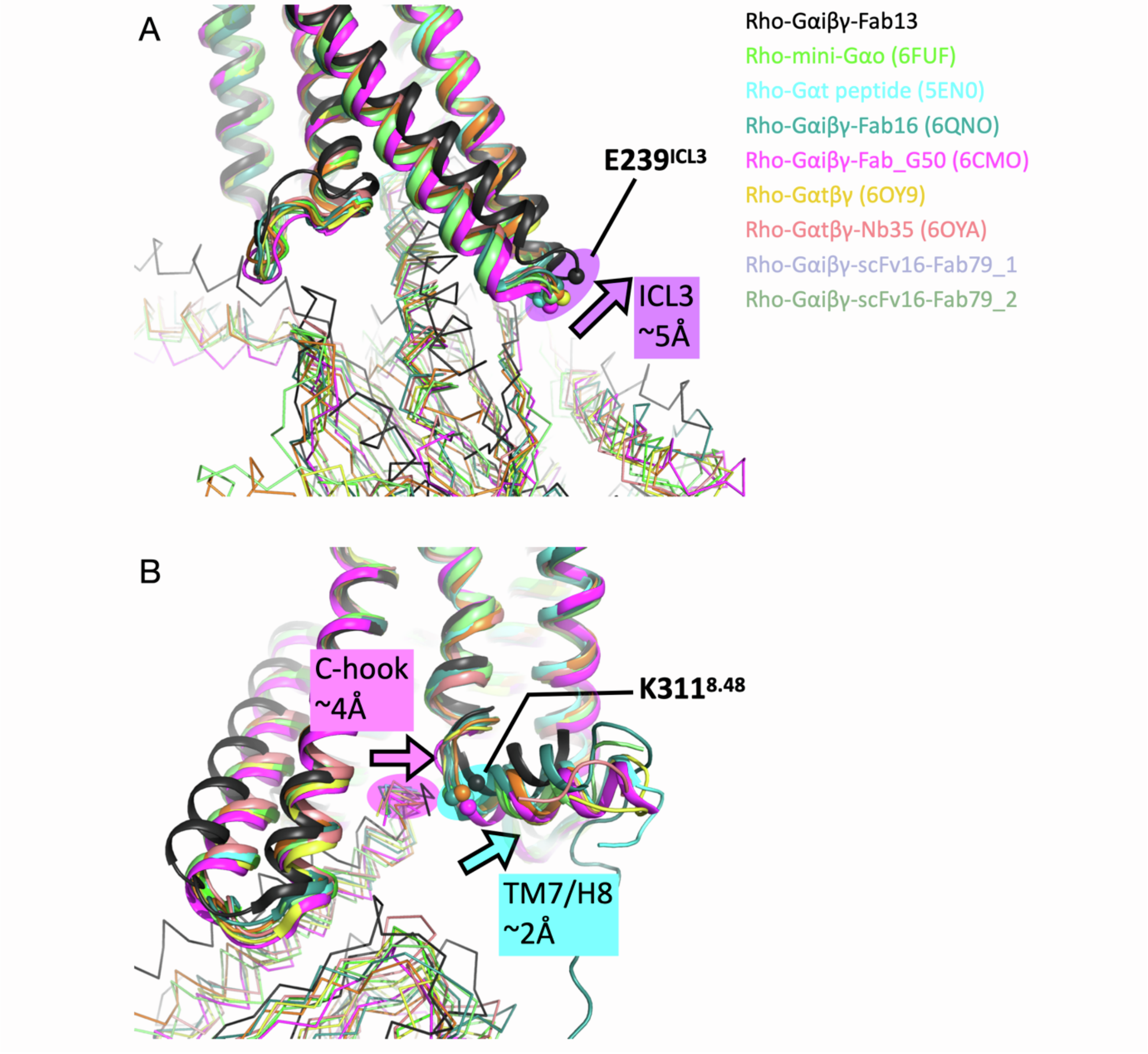

Figure S10. Comparison of Rho-Gαiβγ-Fab13 with other rhodopsin-G protein complexes: ICL3 and TM7/H8 regions. Rhodopsin and Gα are shown as cartoon and ribbon, respectively. The Rho-Gαiβγ-Fab13 structure is colored in black. **(A)** The ICL3 is highlighted with a violet patch. In Rho-Gi-Fab13, ICL3 shows a ~5-Å outward displacement. The Cα atom of E239 is displayed as a sphere in all rhodopsin structures. **(B)** The TM7/H8 turn of rhodopsin and the C-terminal hook (C-hook) of Gα α5 helix are highlighted in cyan and magenta, respectively. The TM7/H8 turn in Rho-Gαiβγ-Fab (black) is shifted outwards by ~2 Å, resulting in a ~4 Å deeper insertion of the C-hook. The Cα atom of $K311^{8.48}$ is displayed as a sphere in all the rhodopsin structures.

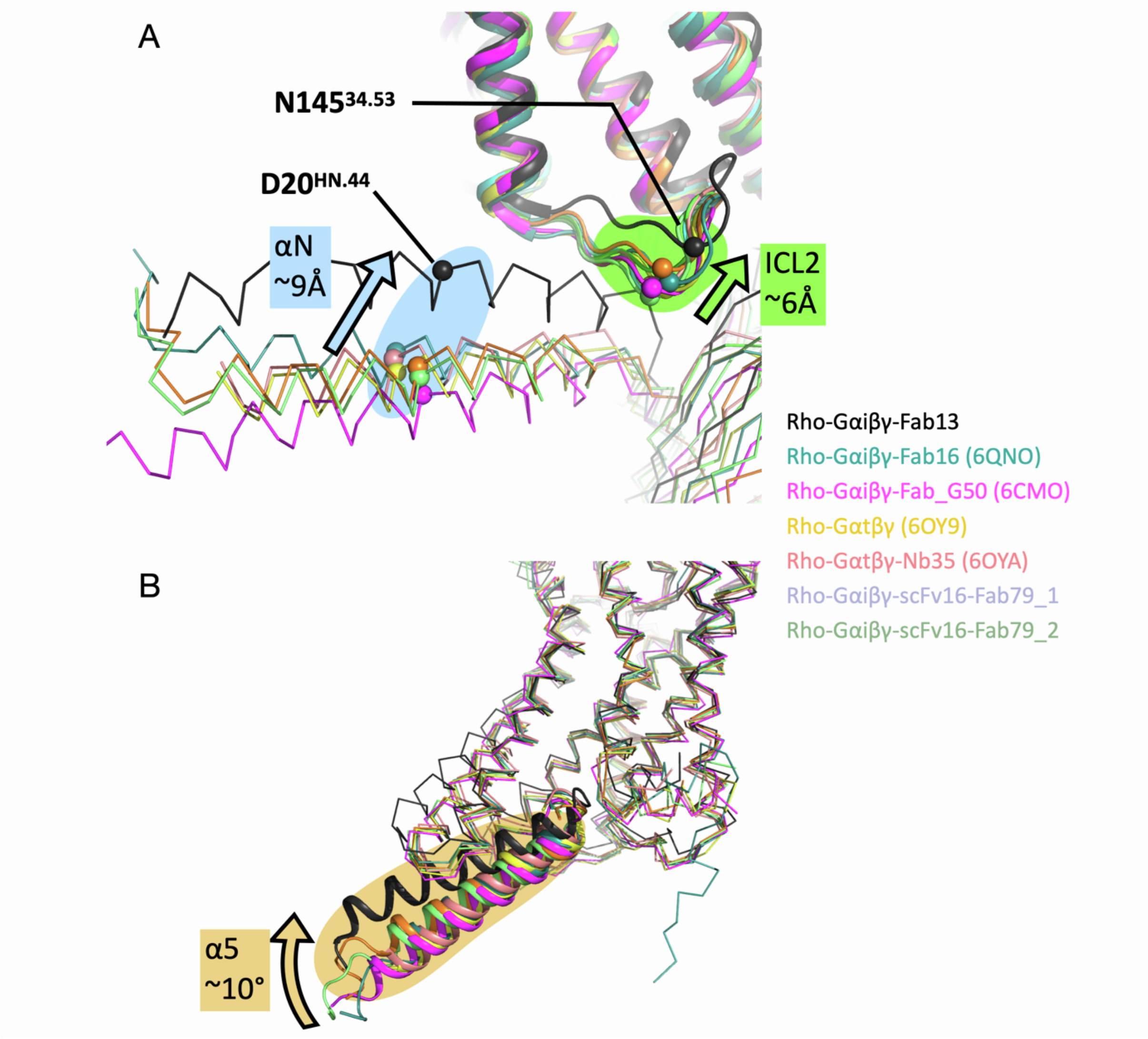

Figure S11. Comparison of Rho-Gαiβγ-Fab13 with other rhodopsin-G protein complexes: ICL2. Rhodopsin and Gα are shown in cartoon and ribbon, respectively. Rho-Gαiβγ-Fab13 is colored in black. **(A)** ICL2 is highlighted with a green patch. The Cα of N145$^{34.53}$ is shown as a sphere. In Rho-Gαiβγ-Fab13, ICL2 exhibits a ~6-Å upward shift, which appears to guide a corresponding ~9 Å upward shift of the αN helix, measured at the Cα of G.HN.44 (Gαi: D20, Gαt: E16; shown as spheres). **(B)** The α5 helix is highlighted with an orange patch. It shows an additional ~10° twist towards the putative membrane plane compared to other structures.

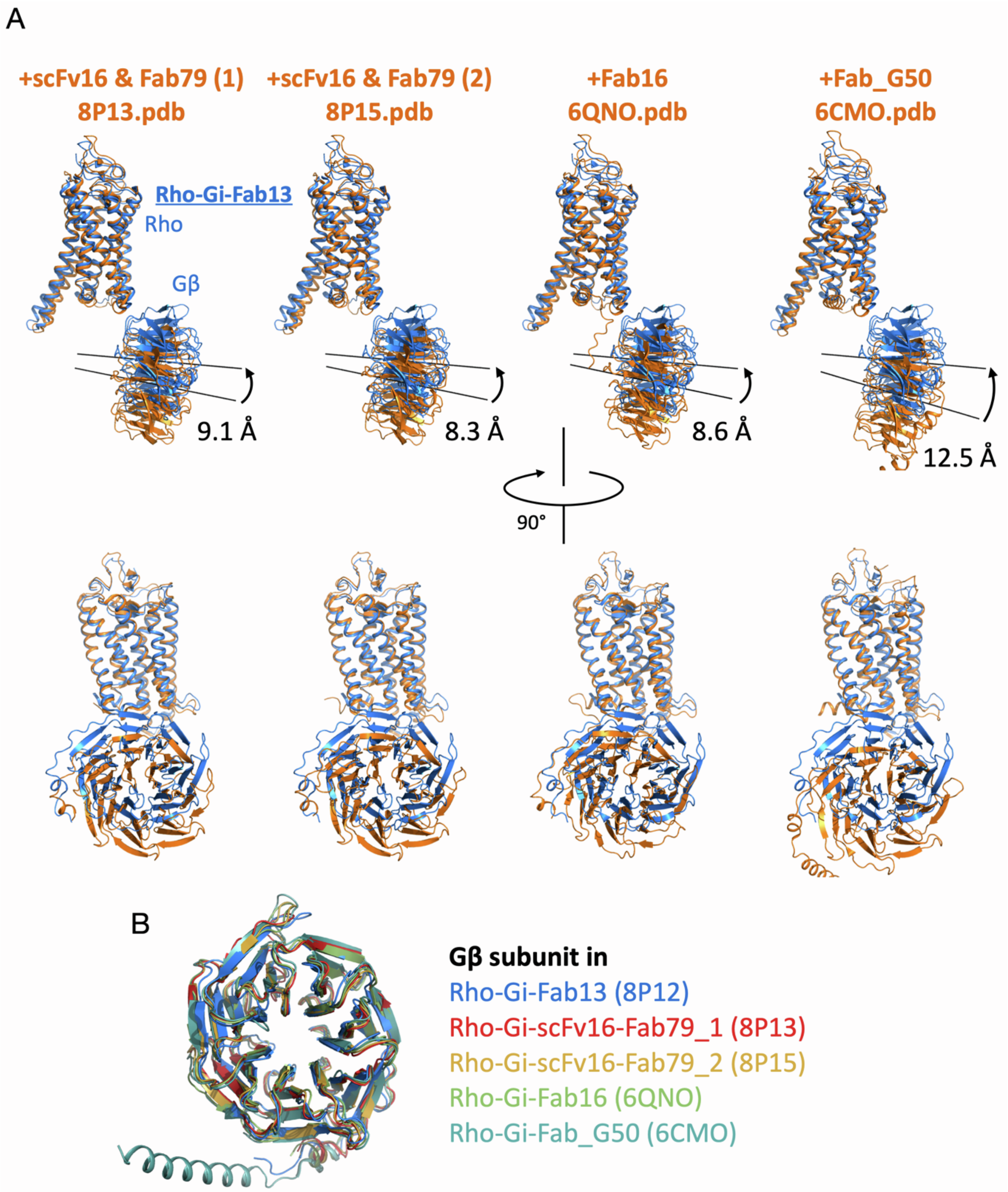

Figure S12. Repositioning of Gβ in the Rho-Gαiβγ-Fab13 complex. (A) Comparison of Gβ position in Rho-Gαiβγ complexes bound to scFv16 and Fab79 (left two panels), Fab16 (middle-right panel), and Fab_G50 (right panel). Structures are aligned to rhodopsin Cα atoms in Rho-Gαiβγ-Fab13. Rhodopsin and Gβ from Rho-Gαiβγ-Fab13 are shown in blue, compared structures in orange. Black lines indicate Gβ central axes. **(B)** Overlay of Gβ subunits aligned to the Cα atoms of Gβ from Rho-Gαiβγ-Fab13.

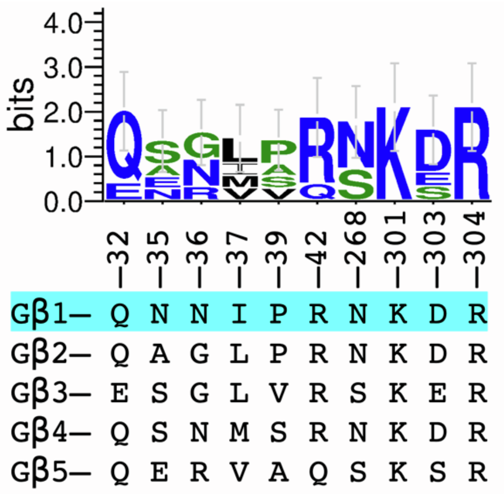

Figure S13. Sequence comparison for Gβ subtypes at the Fab13 binding site. Sequence alignment of the residues in Gβ subtypes 1-5 at residues that form the Fab13 binding interface. Residue conservation and diversity are represented using a Weblogo chart (2).

Fabs	Sequence
**Fab13**	>Fab13_HeavyChain<br>MAVLVLFLCLVAFPSCVLSQVQLKESGPGLVAPSQSLSITCTVSGFSLTNYGVHWVRQPPGKGLEWLGIIWAGGGTSYDSA<br>LMSRLSISKDNSKSQVFLKMNSLQSTDTAMYYCASENYSYDRGFAYWGQGTLVTVSAAKTTPPSVYPLAPGSAAQTNSMVT<br>LGCLVKGYFPEPVTVTWNSGSLSSGVHTFPAVLQSDLYTLSSSVTVPSSTWPSETVTCNVAHPASSTKVDKKIVPRDCGCK<br>PCICTVPEVSSVFIF<br><br>>Fab13_LightChain<br>MKLPVRLLVLMFWIPASSSDVLMTQTPLSLPVSLGDQASISCRSSQIIVNRNGNTYLEWYLQKPGQSPKLLIYKVSNRFSG<br>VPDRFSGSGSGTDFTLKISGVEAEDLGVYYCFQGSHVPWTFGGGTQLEIKRADAAPTVSIFPPSSEQLTSGGASVVCFLNN<br>FYPKDINVKWKIDGSERQNGVLNSWTDQDSKDSTYSMSSTLTLTKDEYERHNSYTCEATHKTSTSPIVKSFNRNEC
**Fab79**	>Fab79_HeavyChain<br>MNFVLSLIFLALILKGVQCEVQLVESGGGLVKPGGSLKLSCAASGFTFSSYAMSWVRQTPEKRLEWVATISSRGLYTYFPD<br>SMKGRFTISRDNAKNTLSLQMSSLRSEDTAMYYCLRGGGYDADYWGQGTTLTVSSAKTTAPSVYPLAPVCGDTTGSSVTLG<br>CLVKGYFPEPVTLTWNSGSLSSGVHTFPAVLQSDLYTLSSSVTVTSSTWPSQSITCNVAHPASSTKVDKKIEPRGPTIKPC<br>PPCKCPAPNLLGGPSVFIF<br><br>>Fab79_LightChain<br>MMSPAQFLFLLVLWIRETNGDVVMTQTPLTLSVTIGQPASISCKSSQSLLDSDGETSLNWLLQRPGQSPKRLIYLVSKLDS<br>GVPDRFTGSGSGTDFTLKISRVEAADLGVYYCWQGTHFPLTFGAGTKLELKRADAAPTVSIFPPSSEQLTSGGASVVCFLN<br>NFYPKDINVKWKIDGSERQNGVLNSWTDQDSKDSTYSMSSTLTLTKDEYERHNSYTCEATHKTSTSPIVKSFNRNEC

Table S1. Protein sequences of Fab13 and Fab79.

Model	Rho-Gi-scFv16-Fab79 (Conformation 1)	
Composition (#)		
Chains	7	
Atoms	12902 (Hydrogens: 0)	
Residues	Protein: 1660	
Water	0	
Ligands	0	
Bonds (RMSD)		
Length (Å) (# > 4σ)	0.005 (0)	
Angles (°) (# > 4σ)	1.129 (26)	
MolProbity score	2.32	
Clash score	25.14	
Ramachandran plot (%)		
Outliers	0.37	
Allowed	6.23	
Favored	93.40	
Rama-Z (Ramachandran plot Z-score, RMSD)		
whole (N = 1637)	-1.00 (0.19)	
helix (N = 417)	-0.59 (0.21)	
sheet (N = 414)	0.04 (0.24)	
loop (N = 806)	-1.01 (0.22)	
Rotamer outliers (%)	0.00	
Cβ outliers (%)	0.00	
Peptide plane (%)		
Cis proline/general	9.8/0.1	
Twisted proline/general	0.0/0.1	
CaBLAM outliers (%)	2.79	
ADP (B-factors)		
Iso/Aniso (#)	12902/0	
min/max/mean		
Protein	164.21/1066.20/474.72	
Nucleotide	---	
Ligand	---	
Water	---	
Occupancy		
Mean	1.00	
occ = 1 (%)	99.50	
0 < occ < 1 (%)	0.05	
occ > 1 (%)	0.00	
**Data**		
Box		
Lengths (Å)	96.50, 122.12, 197.27	
Angles (°)	90.00, 90.00, 90.00	
Supplied Resolution (Å)	5.2	
Resolution Estimates (Å)	Masked	Unmasked
d FSC (half maps; 0.143)	---	2.0
d 99 (full/half1/half2)	3.7/10.1/10.1	3.6/10.2/10.2
d model	3.2	3.3
d FSC model (0/0.143/0.5)	3.2/4.4/7.2	3.3/4.5/7.6
Map min/max/mean	-0.01/0.03/0.00	
**Model vs. Data**		
CC (mask)	0.72	
CC (box)	0.75	
CC (peaks)	0.29	
CC (volume)	0.70	
Mean CC for ligands	---	

Table S2. Model and map statistics of Rho-Gαiβγ-scFv16-Fab79 conformation 1.

**Model**	**Rho-Gi-scFv16-Fab79 (Conformation 2)**	
Composition (#)		
Chains	7	
Atoms	12207 (Hydrogens: 0)	
Residues	Protein: 1572	
Water	0	
Ligands	0	
Bonds (RMSD)		
Length (Å) (# > 4σ)	0.005 (0)	
Angles (°) (# > 4σ)	1.168 (27)	
MolProbity score	2.27	
Clash score	23.74	
Ramachandran plot (%)		
Outliers	0.39	
Allowed	5.73	
Favored	93.89	
Rama-Z (Ramachandran plot Z-score, RMSD)		
whole (N = 1555)	-0.93 (0.20)	
helix (N = 438)	-0.84 (0.21)	
sheet (N = 377)	0.26 (0.25)	
loop (N = 740)	-0.85 (0.23)	
Rotamer outliers (%)	0.00	
Cβ outliers (%)	0.00	
Peptide plane (%)		
Cis proline/general	10.3/0.0	
Twisted proline/general	0.0/0.1	
CaBLAM outliers (%)	2.21	
ADP (B-factors)		
Iso/Aniso (#)	12207/0	
min/max/mean		
Protein	153.16/1055.50/477.82	
Nucleotide	---	
Ligand	---	
Water	---	
Occupancy		
Mean	1.00	
occ = 1 (%)	99.95	
0 < occ < 1 (%)	0.05	
occ > 1 (%)	0.00	
**Data**		
Box		
Lengths (Å)	103.33, 132.37, 193.86	
Angles (°)	90.00, 90.00, 90.00	
Supplied Resolution (Å)	5.9	
Resolution Estimates (Å)	Masked	Unmasked
	---	---
d FSC (half maps; 0.143)	4.1/9.7/9.7	4.1/9.8/9.8
d 99 (full/half1/half2)	3.9	3.8
d model	3.8/4.0/6.9	3.8/4.1/7.3
d FSC model (0/0.143/0.5)	-0.02/0.03/0.00	
Map min/max/mean		
**Model vs. Data**		
CC (mask)	0.72	
CC (box)	0.73	
CC (peaks)	0.30	
CC (volume)	0.70	
Mean CC for ligands	---	

Table S3. Model and map statistics of Rho-Gαiβγ-scFv16-Fab79 conformation 2.

**Model**	**Rho-Gi-Fab13**	
Composition (#)		
Chains	6	
Atoms	10267 (Hydrogens: 0)	
Residues	Protein: 1316	
Water	0	
Ligands	0	
Bonds (RMSD)		
Length (Å) (# > 4σ)	0.008 (3)	
Angles (°) (# > 4σ)	1.109 (20)	
MolProbity score	2.00	
Clash score	17.20	
Ramachandran plot (%)		
Outliers	0.00	
Allowed	3.93	
Favored	96.07	
Rama-Z (Ramachandran plot Z-score, RMSD)		
whole (N = 1301)	-0.37 (0.23)	
helix (N = 360)	0.09 (0.26)	
sheet (N = 341)	0.60 (0.28)	
loop (N = 600)	-0.90 (0.26)	
Rotamer outliers (%)	0.09	
Cβ outliers (%)	0.00	
Peptide plane (%)		
Cis proline/general	11.1/0.0	
Twisted proline/general	0.0/0.0	
CaBLAM outliers (%)	1.80	
ADP (B-factors)		
Iso/Aniso (#)	10267/0	
min/max/mean		
Protein	46.08/588.46/153.53	
Nucleotide	---	
Ligand	---	
Water	---	
Occupancy		
Mean	1.00	
occ = 1 (%)	99.88	
0 < occ < 1 (%)	0.12	
occ > 1 (%)	0.00	
**Data**		
Box		
Lengths (Å)	100.10, 115.70, 141.70	
Angles (°)	90.00, 90.00, 90.00	
Supplied Resolution (Å)	3.2	
Resolution Estimates (Å)	Masked	Unmasked
d FSC (half maps; 0.143)	3.2	3.3
d 99 (full/half1/half2)	3.9/3.3/3.3	3.9/2.9/2.9
d model	3.4	3.3
d FSC model (0/0.143/0.5)	3.1/3.2/4.0	3.2/3.2/4.1
Map min/max/mean	-0.57/1.42/0.03	
**Model vs. Data**		
CC (mask)	0.68	
CC (box)	0.75	
CC (peaks)	0.55	
CC (volume)	0.67	
Mean CC for ligands	--	

Table S4. Model and map statistics of Rho-Gαiβγ-Fab13.

Supporting Citations

1. Cardone, G., J.B. Heymann, and A.C. Steven. (2013). One number does not fit all: mapping local variations in resolution in cryo-EM reconstructions. *J Struct Biol*. 184:226–36.
2. Crooks, G.E., G. Hon, J.-M.M. Chandonia, and S.E. Brenner. (2004). WebLogo: a sequence logo generator. *Genome Res*. 14:1188–1190.
